# Biochemical characterizations of leaves and fruits in *Crataegus monogyna* Jacq., *C. pontica* K.Koch, *C. microphylla* K.Koch, and *C. pentagyna* Waldst. & Kit. ex Willd

**DOI:** 10.1371/journal.pone.0352757

**Published:** 2026-07-06

**Authors:** Sama Koliaei, Javad Erfani-Moghadam, Mehdi Saidi, Ali Khadivi, Yazgan Tunç

**Affiliations:** 1 Department of Horticultural Sciences, Faculty of Agriculture and Natural Resources, Ilam University, Ilam, Iran; 2 Department of Horticultural Sciences, Faculty of Agriculture and Natural Resources, Arak University, Arak, Iran; 3 Republic of Türkiye, Ministry of Agriculture and Forestry, General Directorate of Agricultural Research and Policies, Hatay Olive Research Institute Directorate, Hassa Station, Hassa, Hatay, Türkiye; Institute for Biological Research, University of Belgrade, SERBIA

## Abstract

The genus *Crataegus* comprises a diverse group of species with significant medicinal and nutritional value. This study aimed to characterize the biochemical composition of *Crataegus monogyna* Jacq., *C. pontica* K.Koch, *C. microphylla* K.Koch*,* and *C. pentagyna* Waldst. & Kit. ex Willd fruits and leaves by evaluating their phenolic profiles, antioxidant capacities, and metabolic interactions. The study also assessed biochemical variations among the analyzed samples and the impact of different ecological conditions on biochemical traits. Descriptive statistical analysis revealed substantial variability in phenolic contents among species. The highest coefficients of variation were observed in ferulic acid (200.00%), flavonol (141.40%), and epicatechin (122.75%). Correlation matrix analysis (CMA) demonstrated strong positive relationships between fruit total phenol and fruit total flavonoid (*r* = 0.97*) and between fruit hydroxycinnamic acid and fruit ortho-diphenol (*r* = 1.00**), suggesting possible co-accumulation patterns among these metabolites within the analyzed dataset. Exploratory multiple regression analysis (MRA) indicated statistical associations between antioxidant capacity and several phenolic-related variables, including hydroxycinnamic acid, ortho-diphenol, and cinnamic acid within the analyzed dataset. Principal component analysis (PCA) revealed that the first three principal components (PCs) collectively explained 100.00% of the total variance, with PC1 accounting for 71.00%, PC2 for 19.91%, and PC3 for 9.09% of the variation. PC1 was primarily driven by hydroxycinnamic acid, total flavonoid, and total phenol, indicating their important contribution to the observed biochemical separation among the analyzed samples. PCA grouped *C. monogyna* and *C. pontica* closely together, whereas *C. pentagyna* exhibited a distinct profile, particularly in total phenol, total flavonoid, and hydroxycinnamic acid accumulation. Heat map analysis (HMA) classified the species and biochemical variables into distinct clusters, with ‘*C. pentagyna*’ exhibiting a unique metabolic profile, particularly in total phenol, total flavonoid, and hydroxycinnamic acid accumulation. These findings suggest potential biochemical associations between fruit and leaf phenolics, emphasizing the impact of genetic and ecological factors on phenolic metabolism in *Crataegus* species. The observed associations may provide preliminary information for future breeding-oriented studies aimed at enhancing bioactive compound content for functional food and medicinal applications. Further research integrating transcriptomic and enzymatic analyses is necessary to elucidate the regulatory mechanisms underlying phenolic biosynthesis and environmental adaptability.

## 1. Introduction

The hawthorn berry (*Crataegus*) is a genus comprising over 1000 species, classified within the subfamily Maloideae of the Rosaceae family, and is extensively distributed across Asia and Europe [1]. The *Crataegus* genus is an important group of fruit-bearing plants that has been utilized for centuries due to its diverse applications, including its role in traditional medicine, food production, and ecological benefits [2]. Native to temperate regions, *Crataegus* species are widely cultivated and naturally distributed in various environments, showing remarkable adaptability to different soil and climatic conditions [3]. In recent years, interest in these species has increased, particularly due to their rich biochemical composition, which includes various phenolic compounds associated with plant defense mechanisms, stress tolerance, and postharvest quality.

Phenolic compounds are secondary metabolites that contribute to plant resistance against environmental stresses such as pathogens, UV radiation, and oxidative stress [4]. They also play a critical role in determining fruit quality, affecting parameters such as color, astringency, and storage potential. Among *Crataegus* species, *C. monogyna* Jacq., *C. pontica* K.Koch, *C. microphylla* K.Koch*,* and *C. pentagyna* Waldst. & Kit. ex Willd have gained attention due to their high total phenol, flavonoid, anthocyanin, ortho-diphenol, hydroxycinnamic acid, and flavonol contents, which contribute to their antioxidant properties [5]. These bioactive compounds influence not only the plant’s physiological responses but also its potential applications in food processing and plant-based products [6].

Beyond their total phenolic and flavonoid content, *Crataegus* species contain various individual phenolic compounds, each playing a role in plant metabolism and contributing to fruit and leaf quality [7]. Chlorogenic acid is involved in plant stress response and enzymatic browning [8], while epicatechin contributes to fruit color development and astringency [9]. *p*-coumaric acid and ferulic acid are known for their structural roles in plant cell walls, providing resistance against environmental stressors [10]. Additionally, rutin, quercetin-3-O-glucoside, procyanidin, cinnamic acid, kaempferol-3-O-glucoside, and apigenin are important flavonoids influencing fruit pigmentation, postharvest longevity, and plant-pathogen interactions [11]. Understanding the distribution of these compounds in different *Crataegus* species and plant parts is essential for optimizing fruit utilization, breeding programs, and postharvest management strategies [12].

Although *Crataegus* species have been studied for their biochemical properties, comparative evaluations across different species, particularly in both leaves and fruits, are relatively limited [13]. While some studies have concentrated on specific species or isolated compounds, there is a need for broader biochemical characterizations that encompass multiple phenolic components [14,15]. Examining these variations can offer valuable insights into biochemical variation among sampled *Crataegus* materials, fruit quality attributes, and their potential applications in the food and plant-based industries.

This study aims to characterize the biochemical profiles of the leaves and fruits of *C. monogyna*, *C. pontica*, *C. microphylla*, and *C. pentagyna* by analyzing their total phenolic content, flavonoids, antioxidant capacity, anthocyanins, ortho-diphenols, hydroxycinnamic acids, flavonols, and individual phenolic compounds. Additionally, multivariate statistical analyses will be used to identify biochemical similarities and differences among species, providing a more comprehensive understanding of their metabolic diversity. The findings will contribute to horticultural, postharvest, and breeding research, supporting future applications of *Crataegus* species in fruit production, quality enhancement, and sustainable agriculture.

Given that each Crataegus species was represented by samples collected from a single geographic location, the present study should be considered an exploratory biochemical characterization rather than a definitive species-level comparison.

## 2. Materials and methods

### 2.1. Plant material

**Fig 1:**
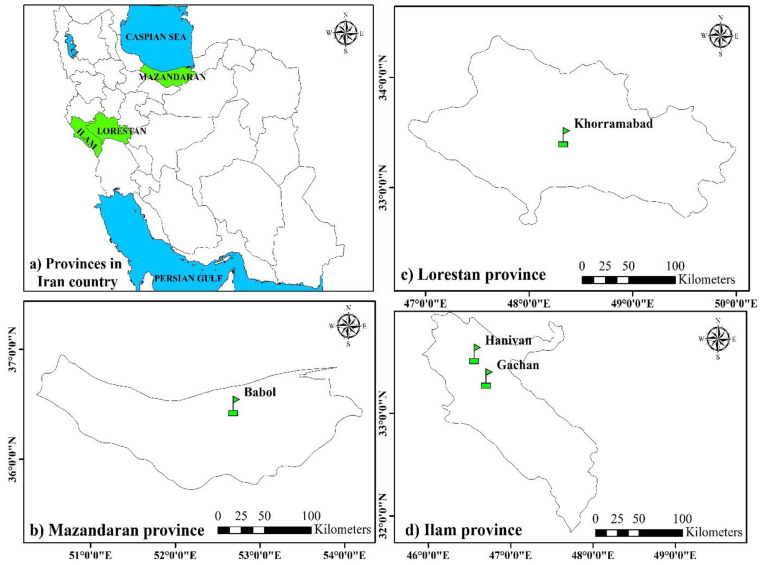
Geographic locations of collection sites of the four studied species of *Crataegus* genus. The map was generated by the fourth author, Ali Khadivi, using ArcGIS software v10.1 and is original; no third-party copyrighted or proprietary material was used, and no permission is required. (ArcGIS: http://www.arcgis.com/home/item.html?id=30e5fe3149c34df1ba922e6f5bbf808f).

Chemical properties of four species of *Crataegus* genus in Iran, including *C. monogyna* (collected from Dorood area of Lorestan province), *C. pontica* (collected from Hanivan area of Ilam province), *C. microphylla* (collected from Gachan area of Ilam province), and *C. pentagyna* (collected from Babol area of Mazandaran province), were evaluated. The geographic locations of collection sites of the studied accessions are shown in Fig 1, generated using ArcGIS software v10.1.

The formal identification of the specimens was performed by Dr. Javad Erfani-Moghadam.

### Statement specifying permissions

For this study, we acquired permission to study *Crataegus* species issued by the Agricultural and Natural Resources Ministry of Iran.

### Statement on experimental research and field studies on plants

The either cultivated or wild-growing plants sampled comply with relevant institutional, national, and international guidelines and domestic legislation of Iran.

Because each species was sampled from a single geographic location, species identity and environmental conditions may not be completely independent. Therefore, the present dataset primarily reflects sample-level biochemical variation under the specific ecological conditions of the collection sites.

For each *Crataegus* species, fruits and leaves were collected from five healthy and mature individual plants growing within the same natural population. The five plants were considered biological replicates. For each biological replicate, three independent methanolic extracts were prepared separately, resulting in a total of fifteen extracts per species. All biochemical analyses were subsequently performed using each extract in triplicate (technical replicates). Therefore, the reported values represent measurements obtained from five biological replicates, three independent extracts per biological replicate, and three technical replicates for each analytical determination.

After collection, leaves and fruits were carefully cleaned to remove foreign materials and damaged tissues. The samples were dried separately at room temperature under shade conditions with continuous airflow and protected from direct sunlight. The dried materials were ground into fine particles using a household grinder and passed through a 40-mesh sieve. To minimize oxidative degradation of phenolic compounds during and after grinding, the powdered samples were immediately transferred into airtight amber glass containers protected from light and air exposure. The powdered samples were stored at −20 °C for no longer than 72 h before extraction and were subjected to methanolic extraction as soon as possible after grinding to minimize oxidative degradation of phenolic compounds.

### 2.2. Chemical evaluations

**Preparation of extracts:** Methanolic extract was prepared according to the method of Liu et al. [16]. For this purpose, one g of ground samples was transferred individually into a 25 ml volumetric flask containing 15 ml of 70% methanol. To increase the solubility of phenolic compounds in the solvent, the samples were placed in an ultrasonic device for 30 minutes, and the obtained extract was passed through filter paper to separate the solid part. Finally, 70% methanol was used to bring the volume of the solution to 25 ml. Then, one ml of the crude extract was diluted with 9 ml of 70% methanol, which is called diluted extract. All extracts were stored in amber glass containers at 4 ˚C and analyzed within 24 h after extraction to minimize oxidative and biochemical degradation of phenolic compounds.

**Total phenol content:** The total phenol content in the diluted extract was measured using the method of Singleton and Rossi [17] using Folin-Ciocalteu reagent. In this method, 50 µL of diluted extract was mixed with 750 µL of Folin-Ciocalteu reagent (diluted with water at a ratio of 1:14). After 3 minutes, 200 µL of 20% sodium bicarbonate was added to provide alkaline conditions necessary for color development. The solution was homogenized and heated for one minute at 100°C in a Bain-Marie and then placed in the dark for 30 minutes to allow completion of the reaction. Finally, the absorbance of the resulting solution was read at a wavelength of 685 nm using a spectrophotometer (ONDA VIS-10 Plus, VIS 325–1000 nm, ONDA Laboratori s.r.l., Perugia, Italy). To prepare the control solution, all steps of the procedure were performed, with the difference that instead of 50 µL of diluted extract, 50 µL of 70% methanol was used. Before reading the samples, the spectrophotometer was zeroed with a blank, and 70% methanol was used as a blank. Although the heating step followed the original analytical protocol, it may have contributed to partial degradation of thermolabile phenolic compounds. Therefore, the obtained values should be interpreted as estimates of total reducing phenolic capacity under the applied extraction and analytical conditions. The total phenol content in the extracts was expressed as mg of gallic acid per g of dry weight (mg GAE/g DW).

**Total flavonoid content:** The total flavonoid content in the diluted extract was measured spectrophotometrically according to the method of Zhishen et al. [18] using an aluminum chloride colorimetric assay. Initially, 60 µL of 5% sodium nitrite was added to 1 µL of diluted extract. After 5 minutes, 60 µL of 10% aluminum chloride was added, and the mixture was allowed to react for 6 min. Subsequently, 400 µL of 1 M sodium hydroxide was added, and the reaction mixture was vortexed thoroughly. Finally, deionized distilled water was added to bring the final reaction volume to 2 mL, and the absorbance was measured at 510 nm using a spectrophotometer. For the control solution, the same procedure was followed using 1 mL of 70% methanol instead of the diluted extract. Before measurements, the spectrophotometer was zeroed using a blank containing 70% methanol. Total flavonoid content was expressed as mg of quercetin per g of dry weight (mg QUE/g DW).

**Ortho-diphenol content:** The ortho-diphenol content in the extracted samples was determined according to the method proposed by Obied et al. [19]. The 250 µL of diluted extract was mixed with 250 µL of 5% sodium molybdate in a flask, and after mixing with 70% methanol, the volume was brought to 5 ml. After 15 minutes, the absorbance was measured at a wavelength of 370 nm. The results were expressed in terms of mg of caffeic acid per g of dry weight (mg CAE/g DW).

**Hydroxycinnamic acid content:** The 500 μl of diluted extract was poured into a 10 ml volumetric flask and made up to 10 ml with 2% HCl. Then, the absorbance of the samples was read at a wavelength of 320 nm using 2% HCl as the blank solution. The results were expressed in terms of mg of caffeic acid per g of dry weight (mg CAE/g DW).

**Flavonol content:** The 500 µL of diluted extract was poured into a 10 ml volumetric flask and made up to 10 ml with 2% HCl. Then, the absorbance of the samples was read at a wavelength of 360 nm. Then, the absorbance of the samples was read at a wavelength of 320 nm using 2% HCl as the blank solution. The results were expressed in terms of mg of quercetin per g of dry weight (mg QUE/g DW).


$$\begin{array}{c} \text{A }=\;({\text{A}}_{\lambda \text{ vis}-\text{max}}\;-\;{\text{A}}_{\text{7}\text{0}\text{0}}{)}_{\text{pH }\text{1}.\text{0}}\;-\;({\text{A}}_{\lambda \text{ vis}-\text{max}}\;-\;{\text{A}}_{\text{7}\text{0}\text{0}}{)}_{\text{pH }\text{4}.\text{5}} \end{array}$$ (1)

$$\begin{array}{c} \text{Total anthocyanin content }(\text{mg C3G}/\text{g DW})\;=\;(A\;\times \text{ MW }\times \text{ DF }\times \text{ 1000})/(\varepsilon \;\times \;\text{1}) \end{array}$$ (2)

Where, A = absorbance of diluted sample, A_λ vis-max_ = absorbance of sample at 510 nm wavelength, A_700_ = absorbance of sample at 700 nm wavelength, MW = molecular mass of cyanidin-3-O-glucoside (449.2), DF = sample dilution factor, and ε = 26.900 dm³·mol⁻¹·cm⁻¹ (molar extinction coefficient of cyanidin-3-O-glucoside).

**Antioxidant capacity (radical scavenging using 2,2-Diphenyl-1-picrylhydrazyl (DPPH)):** The DPPH radical scavenging activity of the samples was evaluated by the method proposed by Obied et al. [19] with minor changes. 50 µL of the diluted extracts was added to 3 ml of methanolic DPPH solution (82%), which exhibited a purple color, and poured into a plastic microtube. The mixture was shaken well and kept in a dark environment for 60 minutes (the color gradually changed from purple to reddish and finally to pale yellow). Then, the absorbance of the samples was read at a wavelength of 517 nm compared with the control sample. To prepare the control solution, 50 µL of 70% methanol (instead of the extract) was added to 3 ml of methanolic-aqueous DPPH solution (82%). The 70% methanol was used for the device blank. The percentage of DPPH radical scavenging activity was also calculated according to equation 3.


$$\begin{array}{c} \%\text{Scavenging }=\text{ 100 }\times \;({\text{A}}_{\text{control}}\;-\;{\text{A}}_{\text{sample}})/{\text{A}}_{\text{control}} \end{array}$$ (3)

Where, %Scavenging = inhibition percentage, A_control_ = control absorption rate, and A_sample_ = sample absorption rate.

**Analysis of extracts with HPLC:** Individual phenolic compounds were analyzed using a high-performance liquid chromatography system (Knauer Smartline HPLC, Knauer Wissenschaftliche Geräte GmbH, Berlin, Germany) equipped with a UV–Vis detector (Smartline UV Detector 2500, Knauer Wissenschaftliche Geräte GmbH, Berlin, Germany). Separation was carried out on a Eurospher II 100-5 C18 reverse-phase analytical column (250 × 4.6 mm, 5 μm; Knauer Wissenschaftliche Geräte GmbH, Berlin, Germany) maintained at 25 °C. The mobile phase consisted of solvent A (water containing 0.05% phosphoric acid) and solvent B (acetonitrile). Elution was performed using a gradient program as follows: 0–10 min, 10% B; 10–25 min, 10–25% B; 25–40 min, 25–40% B; 40–50 min, 40–60% B; followed by re-equilibration to the initial conditions. The flow rate was maintained at 0.6 mL min^-1^, and the injection volume was 10 μL.

Detection of phenolic compounds was performed within the wavelength range of 220–400 nm, and chromatograms were evaluated according to the maximum absorption wavelengths of the corresponding standards. Identification and quantification of chlorogenic acid, epicatechin, p-coumaric acid, procyanidin, ferulic acid, rutin, quercetin-3-O-glucoside, cinnamic acid, kaempferol-3-O-glucoside, and apigenin were achieved by comparing retention times and UV spectra with authentic external standards. Calibration curves were prepared using standard solutions at different concentrations and showed good linearity (R² > 0.99).

All analytical standards were of HPLC grade with purity ≥98% and were purchased from Sigma-Aldrich (St. Louis, MO, USA). Before injection, both standards and sample extracts were filtered through 0.22 μm syringe filters and transferred into 2 mL HPLC vials before analysis [21].

### 2.3. Statistical analysis

Analysis of variance (One-way ANOVA) followed by Tukey’s multiple comparison test (*p* < 0.05) was performed to evaluate the variation among accessions based on the traits measured (S1 File) using JMP^®^ Pro 17 software [22]. Pearson (*r*) correlation coefficients were used to determine the relationship among the recorded traits using Origin Pro^®^ 2025b software [23], and significance levels were evaluated at *p* < 0.05 and *p* < 0.01. Principal component analysis (PCA) was applied using Origin Pro^®^ 2025b software to identify the key traits influencing genotype grouping. To enhance the interpretability of the components, the Varimax rotation with the Kaiser Normalization method was employed. This approach clarified the relationships between components, ensuring a more comprehensible and meaningful analysis. Heat map analysis based on Ward’s method and Euclidean distance coefficients using Origin Pro^®^ 2025b software was used to classify accessions and variables. The first and second principal components (PC1/PC2) were used to draw a two-dimensional biplot by determining the distribution of accessions and quantitative variables using Origin Pro^®^ 2025b software. Because the present study included a limited number of sampled materials, PCA was primarily used as an exploratory multivariate visualization tool to describe relationships among variables and samples within the analyzed dataset. Moreover, fruit-related traits were considered dependent variables, and the characteristics affecting these traits were determined using multiple regression analysis (MRA). The MRA was conducted using “stepwise” method of “linear regression analysis” option of SPSS^®^ (SPSS Inc., Chicago, IL, USA) [24,25] statistics. The multivariate analyses performed in the present study should be interpreted as exploratory analytical approaches intended to describe relationships within the analyzed dataset, rather than definitive evidence of species-level biochemical differentiation.

## 3. Results and discussion

### 3.1. Descriptive statistics among accessions

Descriptive statistics for the chemical traits of the four species of *Crataegus* genus are presented in Table 1. The one-way ANOVA (*p < 0.05*) revealed significant differences among the four evaluated species (Tables 2–4). The highest variation was observed in ferulic acid (200.00%), flavonol (141.40%), epicatechin (122.75%), cinnamic acid (115.49%), and apigenin (106.88%). In contrast, the lowest variation was recorded in kaempferol-3-O-glucoside (11.89%), hydroxycinnamic acid (10.23%), ortho-diphenol (6.50%), fruit antioxidant capacity (5.80%), and leaf antioxidant capacity (1.26%). Notably, 9 out of 15 variables (representing 62.5% in total) had coefficients of variation (CVs) greater than 20.00%. The observed value indicates a considerable level of variability among the genotypes assessed [26]. Traits with a coefficient of variation (CV) greater than 20.00% exhibit more pronounced differences among specimens, making them effective indicators for distinguishing accessions, genotypes, cultivars, or species [27]. Additionally, traits with a wider quantitative spectrum tend to show higher CV% values, reflecting a greater potential for selection and improvement [28]. On the other hand, traits with lower CV values are more consistent, demonstrating stability across various genotypes [27]. Our results are in agreement with those of a study on walnuts in Iran, which reported a CV of 64.86% [29].

**Table 1:** Descriptive statistics for the chemical traits of the studied species of *Crataegus* genus.

Trait	Abb.	Unit	Min	Max	Mean	±SD	CV (%)
**Fruit biochemical contents**							
Total phenol	F1	mg GAE/g DW	55.92	200.68	124.02	±59.64	48.09
Total flavonoid	F2	mg QUE/g DW	10.83	176.08	76.99	±70.11	91.06
Antioxidant capacity	F3	%	18.93	21.77	20.42	±1.18	5.80
Total anthocyanin	F4	mg C3G/g DW	0.05	0.41	0.20	±0.17	82.86
Ortho-diphenol	F5	mg CAE/g DW	314.04	1356.42	665.85	±468.87	70.42
Hydroxycinnamic acid	F6	mg CAE/g DW	194.32	460.14	280.28	±122.50	43.71
Flavonol	F7	mg QUE/g DW	1.30	17.47	5.61	±7.93	141.40
**Fruit individual phenolic contents**							
Chlorogenic acid	F8	mg/g DW	0.27	0.49	0.37	±0.10	28.10
Epicatechin	F9	mg/g DW	0.00	0.32	0.13	±0.16	122.75
*p*-coumaric acid	F10	mg/g DW	0.94	1.20	1.07	±0.14	12.72
Procyanidin	F11	mg/g DW	2.71	16.56	7.05	±6.51	92.38
Ferulic acid	F12	mg/g DW	0.00	1.03	0.26	±0.52	200.00
Rutin	F13	mg/g DW	1.27	6.63	3.68	±2.34	63.69
Quercetin-3-O-glucoside	F14	mg/g DW	7.80	10.51	8.52	±1.33	15.56
Cinnamic acid	F15	mg/g DW	0.00	0.37	0.18	±0.21	115.49
Kaempferol-3-O-glucoside	F16	mg/g DW	15.66	20.15	17.27	±2.05	11.89
Apigenin	F17	mg/g DW	0.23	7.49	3.73	±3.98	106.88
**Leaf biochemical contents**							
Total phenol	L1	mg GAE/g DW	166.54	236.42	188.12	±32.51	17.28
Total flavonoid	L2	mg QUE/g DW	123.88	202.38	155.47	±33.72	21.69
Antioxidant capacity	L3	%	21.04	21.61	21.29	±0.27	1.26
Total anthocyanin	L4	mg C3G/g DW	0.12	0.32	0.21	±0.10	49.76
Ortho-diphenol	L5	mg CAE/g DW	2212.70	2550.40	2362.08	±153.57	6.50
Hydroxycinnamic acid	L6	mg CAE/g DW	935.77	1169.38	1028.59	±105.27	10.23
Flavonol	L7	mg QUE/g DW	44.42	63.26	57.52	±8.79	15.28

*Abb* Abbreviations, *Max* Maximum, *Min* Minimum, *± SD* Standard Deviation, *CV* Coefficient of Variation. Data are presented as mean ± SD. Each species was represented by five biological replicates (individual plants). Three independent extracts were prepared from each biological replicate, and all biochemical analyses were performed in triplicate (technical replicates).

**Table 2:** Phytochemical contents of fruits from the studied *Crataegus* species.

Species	Total phenol (mg GAE/g DW)	Total flavonoid (mg QUE/g DW)	Antioxidant capacity (%)	Total anthocyanin (mg C3G/g DW)
*C. microphylla*	127.44^b^	58.75^c^	20.21^c^	0.26^ab^
*C. monogyna*	112.03^c^	62.29^b^	20.76^b^	0.09^bc^
*C. pentagyna*	200.68^a^	176.08^a^	21.77^a^	0.41^a^
*C. pontica*	55.92^d^	10.83^d^	18.93^d^	0.05^c^
**Species**	**Ortho-diphenol** **(mg CAE/g DW)**	**Hydroxycinnamic acid** **(mg CAE/g DW)**		**Flavonol** **(mg QUE/g DW)**
*C. microphylla*	469.29^c^	212.46^c^		1.30^c^
*C. monogyna*	523.66^b^	254.19^b^		2.35^b^
*C. pentagyna*	1356.42^a^	460.14^a^		17.47^a^
*C. pontica*	314.04^d^	194.32^d^		1.30^c^

Means followed by different letters are significantly different according to Tukey’s multiple comparison test (*p < 0.05*).

**Table 3:** Individual phenolic contents of fruits from the studied *Crataegus* species (mg/g DW).

Species	Chlorogenic acid	Epicatechin
*C. microphylla*	0.27^d^	0.04^b^
*C. monogyna*	0.49^a^	0.19^ab^
*C. pentagyna*	0.43^b^	0.32^a^
*C. pontica*	0.30^c^	0.01^b^
**Species**	***p*-coumaric acid**	**Procyanidin**
*C. microphylla*	1.20^a^	6.01^b^
*C. monogyna*	1.18^b^	2.93^c^
*C. pentagyna*	0.97^c^	16.56^a^
*C. pontica*	0.94^d^	2.71^c^
**Species**	**Ferulic acid**	**Rutin**
*C. microphylla*	0.04^b^	2.46^c^
*C. monogyna*	1.03^a^	4.34^b^
*C. pentagyna*	0.01^b^	6.63^a^
*C. pontica*	0.01^b^	1.27^d^
**Species**	**Quercetin-3-O-glucoside**	**Cinnamic acid**
*C. microphylla*	7.89^b^	0.04^b^
*C. monogyna*	7.80^b^	0.36^a^
*C. pentagyna*	10.51^a^	0.37^a^
*C. pontica*	7.89^b^	0.01^b^
**Species**	**Kaempferol-3-O-glucoside**	**Apigenin**
*C. microphylla*	15.94^c^	6.84^b^
*C. monogyna*	15.66^d^	0.23^c^
*C. pentagyna*	20.15^a^	0.34^c^
*C. pontica*	17.33^b^	7.49^a^

Means followed by different letters are significantly different according to Tukey’s multiple comparison test (*p < 0.05*).

**Table 4:** Biochemical contents of leaves from the studied *Crataegus* species.

Species	Total phenol (mg GAE/g DW)	Total flavonoid (mg QUE/g DW)	Antioxidant capacity (%)	Total anthocyanin (mg C3G/g DW)
*C. microphylla*	177.62^b^	140.93^c^	21.04^b^	0.12^b^
*C. monogyna*	166.54^d^	154.71^b^	21.61^a^	0.27^a^
*C. pentagyna*	236.42^a^	202.38^a^	21.41^a^	0.32^a^
*C. pontica*	171.92^c^	123.88^d^	21.09^b^	0.12^b^
**Species**	**Ortho-diphenol** **(mg CAE/g DW)**		**Hydroxycinnamic acid** **(mg CAE/g DW)**	**Flavonol** **(mg QUE/g DW)**
*C. microphylla*	2264.45^c^		935.77^d^	63.26^a^
*C. monogyna*	2420.76^b^		1047.36^b^	61.57^b^
*C. pentagyna*	2550.40^a^		1169.38^a^	44.42^d^
*C. pontica*	2212.70^d^		961.85^c^	60.83^c^

Means followed by different letters are significantly different according to Tukey’s multiple comparison test (*p < 0.05*).

The biochemical composition of *Crataegus* fruits, particularly their phenolic content and antioxidant properties, provides valuable insights into their nutritional and pharmacological potential. The results indicate a considerable variation in the chemical traits among the four studied species, suggesting variation among the sampled *Crataegus* materials in secondary metabolite accumulation (Table 2).

*C. pentagyna* generally exhibited higher levels of total phenol, total flavonoid, antioxidant capacity, ortho-diphenol, hydroxycinnamic acid, and flavonol contents compared with the other species, indicating its strong phytochemical potential. In contrast, lower values for several phytochemical traits were mainly observed in *C. pontica*. The observed variation among species may be associated with genetic background, ecological conditions, and developmental factors. Overall, the results demonstrate that *Crataegus* fruits represent valuable sources of bioactive compounds with potential nutritional and functional importance.

All biochemical values obtained in the present study were expressed according to the units indicated in the corresponding Tables. Since previous studies have reported *Crataegus* biochemical traits using different unit systems, including dry weight- and fresh weight-based expressions, direct numerical comparisons should be interpreted cautiously. Therefore, the literature comparisons in the present study were discussed mainly in terms of general trends rather than strict quantitative equivalence. Yildiz et al. [30] reported that the antioxidant capacity in hawthorn across twenty-two genotypes ranged from 23.13 to 61.59%, while the total phenolic content varied between 277.28 and 310.80 mg GAE/100 g. Additionally, the total flavonoid content ranged from 14.63 to 57.22 mg GAE/100 g. In a separate study, Güzel [31] recorded total phenolic content values ranging from 155.2 to 490.3 mg GAE/100 g, whereas total flavonoid content was documented between 78.7 and 272.6 mg CE/100 g in different hawthorn genotypes. Okatan et al. [32] determined that phenolic compound concentrations spanned from 960 to 3626 μg GAE/g, with antioxidant capacity levels varying from 59.24 to 19.24% across twenty distinct hawthorn genotypes. Similarly, Çalişkan et al. [33] observed that the antioxidant capacity values of hawthorn genotypes ranged between 21.4 and 33.2%. Alirezalu et al. [34] reported that the total phenolic content, total flavonoid content, and antioxidant capacity in hawthorn genotypes ranged from 21.19 to 69.12 mg GAE/g DW, 2.44 to 6.08 mg QUE/g DW, and 0.32 to 1.84 mmol Fe²⁺/g DW, respectively. Furthermore, Salmanian et al. [35] reported that the total anthocyanin content in hawthorn fruit was determined to be 1.94 mg CE/g. Overall, the findings of the present study are comparable to previous research; however, some differences exist. These differences might stem from the genetic material utilized in the experiments and the methodologies applied. The levels of flavonoids, phenolic compounds, and fruit pigments such as anthocyanins and carotenoids in hawthorn fruits are influenced by elevated temperatures and CO_2_ concentrations [34,36].

The wide range observed in the biochemical composition of these fruits highlights the combined genetic and ecological variability among the sampled *Crataegus* species, which originate from different geographical regions. This inherent diversity may have significant implications for their nutritional value, functional food applications, and potential medicinal uses.

The individual phenolic contents of some *Crataegus* genus fruits are presented in Table 3. The individual phenolic composition of *Crataegus* fruits highlights their rich profile of bioactive compounds with potential health benefits. Chlorogenic acid, which ranges from 0.27 (*C. microphylla*) to 0.49 (*C. monogyna*) mg/g DW, is a well-known antioxidant with anti-inflammatory and neuroprotective properties. It has been associated with improved glucose metabolism and reduced risk of type 2 diabetes.

Epicatechin, present in at concentrations of up to 0.32 (*C. pentagyna*) mg/g DW, is a flavonol known for its cardiovascular benefits, particularly in reducing blood pressure and improving endothelial function. The presence of *p*-coumaric acid, ranging from 0.94 (*C. pontica*) to 1.20 (*C. microphylla*) mg/g DW, suggests additional antioxidant and anti-inflammatory potential, as this compound is known to protect against oxidative stress-induced cellular damage.

Procyanidin content, which varies widely from 2.71 (*C. pontica*) to 16.56 (*C. pentagyna*) mg/g DW, is particularly noteworthy due to its strong antioxidant capacity and potential role in reducing the risk of cardiovascular diseases by improving vascular health. Ferulic acid, detected in some samples at concentrations up to 1.03 (*C. monogyna*) mg/g DW, is another potent antioxidant with reported benefits in neuroprotection and skin health.

Rutin, which ranges from 1.27 (*C. pontica*) to 6.63 (*C. pentagyna*) mg/g DW, is a well-known flavonoid with vascular protective effects, including strengthening capillaries and reducing inflammation. Quercetin-3-O-glucoside, found at relatively high levels between 7.80 (*C. monogyna*) and 10.51 (*C. pentagyna*) mg/g DW, has been widely studied for its anti-inflammatory, anticancer, and antihypertensive properties.

Cinnamic acid, detected at levels up to 0.37 (*C. pentagyna*) mg/g DW, has antimicrobial properties and plays a role in metabolic regulation. Kaempferol-3-O-glucoside, which ranges from 15.66 (*C.* *monogyna*) to 20.15 (*C. pentagyna*) mg/g DW, is one of the most abundant phenolic compounds in *Crataegus* fruits. It is known for its antioxidant, anti-inflammatory, and potential anticancer effects. Apigenin, present in concentrations ranging from 0.23 (*C. monogyna*) to 7.49 (*C. pontica*) mg/g DW, has been linked to neuroprotection, anxiety reduction, and anti-inflammatory activity.

A study conducted in Portugal reported that epicatechin ranged from 1.84 to 5.19 mg/kg DW [37], while in Iran, chlorogenic acid ranged from 0.06 to 1.16 mg/kg DW, Vitexin-2-O-rhamnoside from 0.08 to 0.17 mg/kg DW, Vitexin from 0.06 to 0.31 mg/kg DW, rutin from 0.12 to 2.68 mg/kg DW, hyperoside from 0.87 to 2.94 mg/kg DW, isoquercetin from 0.24 to 1.59 mg/kg DW, and quercetin from 0.03 to 0.06 mg/kg DW [34]. In Türkiye, aminobenzoic acid ranged from 10.46 to 38.56 mg/kg DW, chlorogenic acid from 1.02 to 16.16 mg/kg DW, epicatechin from 2.06 to 13.83 mg/kg DW, p-coumaric acid from 0.24 to 7.43 mg/kg DW, ferulic acid from 0.29 to 5.06 mg/kg DW, and rutin from 1.57 to 74.05 mg/kg DW [7]. Muradoğlu et al. [38] examined the phenolic composition of the fruits of *C. monogyna* subsp. *monogyna* Jacq., *C. atrosanguinea* Pojark., *C. orientalis* var. *orientalis* Pallas ex Bieb., and *C. meyeri* Pojark., identifying a diverse range of individual phenolic compounds. The concentrations of these compounds varied considerably, with protocathechuic acid ranging from 2.1 to 4.9 mg/100 g FW, rutin from 23.6 to 61.6 mg/100 g FW, and quercetin from 2.0 to 3.4 mg/100 g FW. Similarly, gallic acid levels spanned from 0.4 to 3.9 mg/100 g FW, while catechin was detected between 17.6 and 54.4 mg/100 g FW. Chlorogenic acid concentrations extended from 2.5 to 7.3 mg/100 g FW, whereas caffeic acid exhibited a broader range, from 19.4 to 81.3 mg/100 g FW. In addition, syringic acid was found between 6.8 and 12.9 mg/100 g FW, *p*-coumaric acid between 3.6 and 6.9 mg/100 g FW, and ferulic acid from 0.9 to 2.9 mg/100 g FW. Furthermore, q-coumaric acid ranged from 0.6 to 0.8 mg/100 g FW, while phloridzin concentrations varied between 0.7 and 4.5 mg/100 g FW. Our findings partially overlap with previous studies. These similarities may be attributed to common environmental factors, genetic variation, and the methods employed in sample collection and analysis. However, discrepancies in the results are also likely due to differences in geographical conditions, as well as variations in plant material and analytical techniques used in each study.

From a health perspective, the diverse phenolic profile of *Crataegus* fruits suggests a significant potential for preventing chronic diseases, particularly those related to oxidative stress and inflammation. The combined effects of these compounds may contribute to cardiovascular health, neuroprotection, and metabolic regulation. The wide variation in phenolic concentrations among different *Crataegus* species is believed to stem from genetic and environmental factors.

The biochemical contents of leaves from the studies *Crataegus* species are presented in Table 4. The biochemical composition of *Crataegus* leaves differs substantially from that of the fruits, both in terms of the concentration and the variability of secondary metabolites. While both organs contain substantial amounts of phenolic compounds, leaves generally exhibit higher concentrations, suggesting greater accumulation of these bioactive compounds in vegetative tissues. This suggests that leaves might play a more significant role in plant defense mechanisms, particularly against herbivory and environmental stressors such as UV radiation and pathogen attacks.

The total phenol content in leaves, ranging from 166.54 (*C. monogyna*) to 236.42 (*C. pentagyna*) mg GAE/g DW, is markedly higher than that in fruits. This difference may be attributed to the fact that leaves are directly exposed to external stressors throughout their lifespan, necessitating a stronger antioxidant system. The total flavonoid content in leaves, which varies between 123.88 (*C. pontica*) and 202.38 (*C. pentagyna*) mg QUE/g DW, also exceeds that of fruits, reinforcing the idea that these compounds play a critical role in protecting photosynthetic tissues from oxidative damage.

Despite the higher phenolic content, the antioxidant capacity of leaves, ranging from 21.04 (*C. microphylla*) to 21.61% (*C. pentagyna*), does not differ substantially from that of fruits. This suggests that while leaves contain more phenolics, not all of these compounds contribute equally to radical-scavenging activity. The specific composition of phenolic compounds, rather than their total amount, might be a more important determinant of antioxidant potential.

One of the most striking differences between leaves and fruits is the level of ortho-diphenol content. The values observed in leaves, between 2212.70 (*C. pontica*) and 2550.40 (*C. pentagyna*) mg CAE/g DW, are significantly higher than those in fruits. This could be linked to the role of these compounds in lignin biosynthesis, which strengthens leaf cell walls and enhances resistance to biotic and abiotic stress. Similarly, hydroxycinnamic acid content in leaves, ranging from 935.77 (*C. pontica*) to 1169.38 (*C. pentagyna*) mg CAE/g DW, is also higher than in fruits. These compounds are well known for their roles in UV protection and antimicrobial defense, further supporting their defensive role in leaves.

Total anthocyanin content in leaves is relatively low, between 0.12 (*C. microphylla*) and 0.32 (*C. pentagyna*) mg C3G/g DW, suggesting that these pigments are less relevant in vegetative tissues compared with those in fruits. This aligns with their primary role in attracting pollinators and seed dispersers in fruit tissues rather than serving as protective agents in leaves. Flavonol content, on the other hand, is significantly elevated in leaves, varying from 44.42 (*C. pentagyna*) to 63.26 (*C. microphylla*) mg QUE/g DW. This is expected, as flavonols are known to accumulate in leaf epidermal cells, where they act as UV filters and protect against photodamage.

Alirezalu et al. [1] reported that the total phenolic content, total flavonoid content, and antioxidant capacity in the leaves of hawthorn genotypes ranged from 12.41 to 82.74 mg GAE/g DW, 3.34 to 9.90 mg/g DW, and 0.21 to 1.16 mmol Fe²⁺/g DW, respectively. Comparable findings have also been reported for total phenolic content, with values of 12.8 mg GAE/g DW for *C. monogyna* [20], 2.9 mg GAE/g DW for *C. pinnatifida* [39], and 26.4 mg GAE/g DW for *C. monogyna* [40]. Our findings, in contrast to previous studies, show significant differences. These discrepancies may be attributed to variations in genetic backgrounds, environmental conditions, and the methodologies employed. Differences in geographic origin, climate factors, and extraction techniques likely contributed to the observed variations in biochemical composition.

The marked biochemical differences between *Crataegus* leaves and fruits highlight the distinct physiological roles of these plant organs. While fruits primarily serve as reproductive structures that attract dispersers, leaves function as metabolically active organs that require robust protective mechanisms. The high phenolic and flavonoid content in leaves suggests their potential for pharmaceutical and nutraceutical applications, particularly as sources of antioxidants and antimicrobial agents. Future studies focusing on the specific phenolic profiles and their bioavailability in different plant parts could provide a more comprehensive understanding of the functional significance of these compounds.

### 3.2. Correlation matrix analysis (CMA)

Simple correlations among biochemical variables in fruits and leaves of the four studied *Crataegus* species are illustrated in Table 5. The strong correlations between fruit total phenol and fruit total flavonoid (*r* = 0.97*), fruit antioxidant capacity (*r* = 0.95*), and fruit total anthocyanin (*r* = 0.95*) suggest a coordinated metabolic regulation among these bioactive compounds. The association between total phenol and total flavonoid indicates their co-regulation via the phenylpropanoid pathway, highlighting their functional interdependence. The correlation with antioxidant capacity reinforces the crucial role of phenolics in oxidative stress defense, while the relationship with total anthocyanin suggests a metabolic link between structural and antioxidative functions.

**Table 5:** Simple correlations among biochemical variables in fruits and leaves of the studied *Crataegus* species.

Variables	F1	F2	F3	F4	F5	F6	F7	L1	L2	L3	L4	L5	L6	L7
F1	1													
F2	0.97*	1												
F3	0.95*	0.93	1											
F4	0.94*	0.90	0.78	1										
F5	0.93	0.99*	0.87	0.87	1									
F6	0.90	0.98*	0.86	0.82	1.00**	1								
F7	0.87	0.95*	0.79	0.83	0.99*	0.99*	1							
L1	0.86	0.93	0.73	0.89	0.97*	0.95*	0.98*	1						
L2	0.95	0.99*	0.95	0.83	0.98*	0.98*	0.95	0.90	1					
L3	0.37	0.44	0.65	0.04	0.41	0.47	0.36	0.18	0.56	1				
L4	0.72	0.80	0.86	0.46	0.80	0.84	0.77	0.63	0.88	0.87	1			
L5	0.86	0.91	0.95	0.65	0.90	0.92	0.85	0.75	0.96*	0.77	0.97*	1		
L6	0.79	0.89	0.84	0.61	0.92	0.95*	0.92	0.82	0.94	0.69	0.95*	0.96*	1	
L7	−0.80	−0.91	−0.72	−0.77	−0.96*	−0.97*	−1.00**	−0.97*	−0.91	−0.33	−0.74	−0.81	−0.91	1

* *p* ≤ 0.05; ** *p* ≤ 0.01. For abbreviations, please see Table 1.

The strong correlations between fruit total flavonoid and multiple biochemical constituents, such as fruit ortho-diphenol (*r* = 0.99*), fruit hydroxycinnamic acid (*r* = 0.98*), fruit flavonol (*r* = 0.95*), and leaf total flavonoid (*r* = 0.99*), indicate a coordinated biosynthetic pathway involving these compounds. These associations suggest that flavonoid metabolism is interconnected with other phenolic derivatives, reinforcing the role of flavonoids in antioxidant defense mechanisms.

Fruit ortho-diphenol exhibited a perfect correlation with fruit hydroxycinnamic acid (*r* = 1.00**), along with strong positive correlations with fruit flavonol (*r* = 0.99*), leaf total phenol (*r* = 0.97*), and leaf total flavonoid (*r* = 0.98*). Ortho-diphenol and hydroxycinnamic acid may share a common biosynthetic pathway or be co-regulated under similar physiological conditions. The relationship between ortho-diphenol and hydroxycinnamic acid is particularly noteworthy, as both are known to be involved in oxidative stress response and plant defense mechanisms.

The strong correlations observed between fruit hydroxycinnamic acid and fruit flavonol (*r* = 0.99), leaf total phenol (*r* = 0.95*), leaf total flavonoid (*r* = 0.98*), and leaf hydroxycinnamic acid (*r* = 0.95*) emphasize the interconnected nature of these compounds across different plant tissues. Hydroxycinnamic acid plays a pivotal role in fruit biochemistry and also influences leaf phenolic composition, potentially reflecting systemic metabolic regulation.

The significant correlation between fruit flavonol and leaf total phenol (*r* = 0.98*) suggests that flavonol compounds in fruits and total phenols in leaves may be functionally linked, possibly due to shared biosynthetic precursors or environmental adaptation mechanisms.

Leaf biochemicals also exhibited notable correlations, with total flavonoid showing a significant relationship with leaf ortho-diphenol (*r* = 0.96*). Leaf total anthocyanin was strongly correlated with leaf ortho-diphenol (*r* = 0.97*) and leaf hydroxycinnamic acid (*r* = 0.95*), indicating potential co-accumulation of these compounds in response to physiological or environmental factors. The correlation between leaf ortho-diphenol and leaf hydroxycinnamic acid (*r* = 0.96*) suggests a biochemical link that may be associated with plant stress responses or enzymatic activity related to phenolic metabolism.

Erkek et al. [7] reported significant positive correlations between total phenols, total flavonoids, antioxidant capacity, and total anthocyanins in a similar study on *Crataegus*. Additionally, our findings are consistent with previous studies that show flavonoids constitute an important subgroup of phenolic compounds and significantly contribute to antioxidant properties [1,41,42]. The observed consistency between our findings and other studies further supports the hypothesis that the accumulation of phenolic compounds in *Crataegus* species is regulated by a coordinated metabolic network. The fact that flavonoids and anthocyanins together enhance antioxidant potential underscores that phenolic metabolism is shaped not only by genetic factors but also by ecological and physiological conditions, highlighting the crucial role of *Crataegus* fruits in determining biologically active properties.

These findings highlight the intricate relationships among phenolic and flavonoid compounds in *Crataegus* fruits and leaves, suggesting possible coordinated accumulation patterns among these metabolites. The observed associations may provide exploratory information for future breeding and selection studies, where enhancing one compound could positively influence the accumulation of others with beneficial bioactive properties. Further investigations, including transcriptomic and enzymatic studies, could provide deeper insights into the regulatory mechanisms governing these associations.

Simple correlations among individual phenolic content variables in fruits of the four studied *Crataegus* species are illustrated in Table 6. The significant positive correlation between chlorogenic acid and cinnamic acid (*r* = 0.96*) suggests a strong biochemical association, likely reflecting their involvement in interconnected biosynthetic pathways. Chlorogenic acid, a major hydroxycinnamic acid derivative, serves as a precursor in phenylpropanoid metabolism, which also gives rise to cinnamic acid. This relationship implies that the accumulation of these compounds is co-regulated, potentially influenced by similar enzymatic activities or environmental factors. Similarly, a highly significant positive correlation was observed between chlorogenic acid and rutin (*r* = 0.99*) and quercetin-3-O-glucoside (*r* = 0.99*), further supporting the idea of a coordinated biosynthetic process. Conversely, a strong negative correlation between chlorogenic acid and apigenin (*r* = −0.96*) suggests that these compounds may follow divergent metabolic routes, where one may inhibit or compete with the accumulation of the other. Together, these findings highlight the complex interplay of phenolic compounds in *Crataegus* species, regulated by both genetic and environmental factors.

**Table 6:** Simple correlations among individual phenolic content variables in fruits of the studied *Crataegus* species.

Variables	F8	F9	F10	F11	F12	F13	F14	F15	F16	F17
F8	1									
F9	0.82	1								
F10	0.08	−0.20	1							
F11	0.24	0.75	−0.37	1						
F12	0.75	0.25	0.53	−0.42	1					
F13	0.74*	0.98*	−0.07	0.81	0.19	1				
F14	0.34*	0.81	−0.51	0.98*	−0.36	0.83*	1			
F15	0.96*	0.94	0.01	0.49	0.56	0.90	0.57	1		
F16	0.17	0.65	−0.77	0.88	−0.52	0.62	0.94	0.37	1	
F17	−0.96*	−0.93	−0.08	−0.48	−0.59	−0.90	−0.54	−1.00*	−0.33	1

* *p* ≤ 0.05; ** *p* ≤ 0.01. For abbreviations, please see Table 1.

The strong correlation between epicatechin and rutin (*r* = 0.98*) indicates that these flavonoid compounds are likely co-synthesized or regulated within the same metabolic network. Epicatechin, a flavan-3-ol, and rutin, a flavonol glycoside, are both known for their antioxidant and anti-inflammatory properties. Their positive association suggests that their biosynthesis may be modulated by shared genetic or enzymatic controls, reinforcing the idea that flavonoid metabolism in *Crataegus* fruits is tightly coordinated.

The significant correlation between procyanidin and quercetin-3-O-glucoside (*r* = 0.98*) further supports the hypothesis of flavonoid co-regulation. Procyanidins are oligomeric flavan-3-ols that contribute to plant defense and pigmentation, while quercetin-3-O-glucoside, a flavonol glycoside, is involved in antioxidant mechanisms. Their close association suggests that both compounds may be regulated by similar physiological or environmental conditions, potentially enhancing fruit quality and bioactivity.

The significant positive correlation between rutin and quercetin-3-O-glucoside (*r* = 0.99*) suggests a very strong biochemical relationship between these two flavonoids. This finding implies that these compounds may share similar biosynthetic pathways or be co-regulated during their synthesis. Both rutin and quercetin-3-O-glucoside are flavonoid glycosides, with quercetin-3-O-glucoside being a glycosylated form of quercetin, while rutin is a rutinose glycoside of quercetin. The observed high correlation indicates that their accumulation might be closely linked, possibly due to shared enzymatic processes or similar precursor molecules within the flavonoid biosynthetic pathway. This close relationship supports the hypothesis that these two compounds are metabolically interconnected, and their synthesis might be regulated by common factors, such as environmental conditions or genetic regulation. Additionally, the strong correlation suggests that any factors influencing the production of one of these flavonoids may also impact the levels of the other.

The perfect negative correlation between cinnamic acid and apigenin (*r* = −1.00**) indicates that these compounds are regulated through opposing biosynthetic pathways. Cinnamic acid is a key precursor in the early steps of the phenylpropanoid pathway and plays a crucial role in the synthesis of various phenolic compounds. This negative relationship suggests that apigenin biosynthesis increases as cinnamic acid levels decrease or follows an inverse regulatory mechanism. This finding highlights the dynamic equilibrium in the production of phenolic compounds in fruit, where certain metabolic pathways are downregulated while others are activated. Apigenin, a flavone compound, is primarily involved in plant defense and stress response mechanisms, whereas cinnamic acid contributes to the synthesis of lignin and other phenolic compounds. The inverse fluctuation of these two compounds suggests that biosynthetic pathways are differentially regulated depending on developmental stages and environmental conditions.

A similar study conducted in China reported significant positive correlations between chlorogenic acid and rutin, chlorogenic acid and quercetin-3-O-glucoside, and between rutin and quercetin-3-O-glucoside [43]. Similar findings were reported in similar studies conducted in Türkiye. [7,33]. These consistent findings across different geographical regions suggest that the biochemical relationships between these compounds may be universally applicable, potentially reflecting shared biosynthetic pathways and metabolic interactions. Our results align with those of previous researchers, reinforcing the idea that chlorogenic acid, rutin, and quercetin-3-O-glucoside are closely related in terms of their accumulation and biosynthesis. This consistency also highlights the robustness of these metabolic connections, which may be influenced by both genetic and environmental factors.

These findings highlight the intricate biochemical relationships governing the accumulation of phenolic and flavonoid compounds in *Crataegus* fruits and leaves, supporting the hypothesis that these metabolites are co-regulated through shared biosynthetic pathways. The strong correlations observed suggest a coordinated regulatory mechanism that ensures the balanced production of key bioactive metabolites. These observed associations may serve as preliminary information for future studies focusing on breeding and biochemical characterization. Further investigations, including transcriptomic and enzymatic studies, are necessary to elucidate the precise genetic and biochemical pathways underlying these interactions. Such studies could provide deeper insights into the regulatory mechanisms governing these associations, potentially aiding in the development of *Crataegus*-based functional foods and medicinal applications.

### 3.3. Multiple regression analysis (MRA)

The MRA results presented in Table 7 reveal the key chemical characteristics associated with medicinal traits in the four studied *Crataegus* species. The significant relationships between dependent and independent variables provide exploratory insights into the statistical relationships among phenolic-related variables in fruits and leaves.

**Table 7:** The chemical characteristics associated with the main medicinal traits in the studied species of *Crataegus* genus, as revealed using MRA and coefficients.

Dependent character	Independent character	*r*	*r* ^*2*^	*β*	*t* value	*p* value
(F) Total phenol	(F) Total flavonoid	0.974^a^	0.95	0.97	6.07	0.03
	(F) Hydroxycinnamic acid	1.000^b^	1.00	1.98	119.32	0.01
	(F) Ortho-diphenol	1.000^c^	1.00	0.90	25.02	0.01
(F) Total flavonoid	(L) Total flavonoid	0.989^a^	0.98	0.99	9.54	0.01
	(L) Total anthocyanin	1.000^b^	1.00	1.26	61.38	0.01
	(F) Cinnamic acid	1.000^c^	1.00	1.03	46.03	0.01
(F) Antioxidant capacity	(F) Rutin	0.969^a^	0.94	0.97	5.52	0.03
(L) Total phenol	(F) Quercetin-3-O-glucoside	0.994^a^	0.99	0.99	12.43	0.01
(L) Total flavonoid	(F) Total flavonoid	0.989^a^	0.98	0.99	9.54	0.01
	(L) Total anthocyanin	1.000^b^	1.00	0.79	61.38	0.01
	(F) Total anthocyanin	1.000^c^	1.00	0.98	27.06	0.01
(L) Antioxidant capacity	(F) Chlorogenic acid	0.996^a^	0.99	1.00	15.98	0.01

*r* Correlation coefficient, *r²* Coefficient of determination, *β* Standardized beta coefficients. *(F)* Fruit, *(L)* Leaf.

Total phenol in fruit is significantly influenced by total flavonoid (*β* = 0.97, *p* = 0.03), hydroxycinnamic acid (*β* = 1.98, *p* = 0.01), and ortho-diphenol (*β* = 0.90, *p* = 0.01). The high standardized beta coefficient for hydroxycinnamic acid suggests that it shows a strong statistical association with total phenol values, likely due to its involvement in the phenylpropanoid pathway. Ortho-diphenol also exhibits a strong association, indicating a possible association with antioxidant-related biochemical traits.

Total flavonoid in fruit is significantly correlated with total flavonoid in leaves (*β* = 0.99, *p* = 0.01), total anthocyanin in leaves (*β* = 1.26, *p* = 0.01), and cinnamic acid in fruit (*β* = 1.03, *p* = 0.01). These results suggest that leaf phenolics showed statistical associations with fruit flavonoid content, suggesting possible coordinated accumulation patterns across plant tissues. The influence of cinnamic acid may reflect its biochemical association with flavonoid-related compounds in flavonoid biosynthesis.

Antioxidant capacity in fruit is significantly influenced by rutin (*β* = 0.97, *p* = 0.03), indicating a possible association with antioxidant activity to radical-scavenging activity. Similarly, leaf total phenol is strongly associated with quercetin-3-O-glucoside in fruit (*β* = 0.99, *p* = 0.01), suggesting a statistical relationship between fruit flavonols and leaf phenolic traits.

Leaf total flavonoid is significantly related to fruit total flavonoid (*β* = 0.99, *p* = 0.01), leaf total anthocyanin (*β* = 0.79, *p* = 0.01), and fruit total anthocyanin (*β* = 0.98, *p* = 0.01). This suggests possible biochemical associations between flavonoid synthesis in fruits and leaves, indicating potential relationships between anthocyanin and flavonoid-related variables.

Leaf antioxidant capacity is highly dependent on fruit chlorogenic acid (*β* = 1.00, *p* = 0.01), suggesting that chlorogenic acid showed a strong statistical association with leaf antioxidant capacity within the analyzed dataset across plant tissues.

Overall, the MRA results revealed several exploratory statistical associations among fruit and leaf biochemical variables in the analyzed *Crataegus* materials. The observed relationships may provide preliminary information for future studies focusing on phenolic metabolism and biochemical characterization. However, considering the limited number of analyzed samples and the exploratory nature of the statistical analyses, these findings should be interpreted cautiously.

Due to the absence of any previous studies conducting MRA using similar variables among *Crataegus* species, the obtained findings were independently evaluated and discussed comparatively within the study itself. This approach enhances the originality of the research and makes a significant contribution towards filling the gap in the existing literature.

MRA has been successfully applied to various fruit species, such as walnut [44], pomegranate [45], and apple [46], to understand the effects of genetic diversity, environmental factors, and cultivation conditions on fruit characteristics.

### 3.4. Principal component analysis (PCA)

The results of the PCA highlight the major contributors to variation in the studied chemical traits of *Crataegus* species (Table 8). PC1 accounted for 71.00% of the total variation, indicating that it is the dominant axis explaining the largest proportion of variability among the analyzed traits. The primary contributors to PC1 included fruit hydroxycinnamic acid (0.24), fruit epicatechin (0.24), fruit rutin (0.24), leaf total flavonoid (0.24), leaf ortho-diphenol (0.24), and leaf hydroxycinnamic acid (0.24). The high factor loadings of these compounds suggest that PC1 primarily represents variations in phenolic composition and antioxidant-related traits, particularly those associated with hydroxycinnamic acids and flavonoids, which are known for their strong antioxidant properties and their role in plant defense mechanisms.

**Table 8:** Eigenvalues of the principal component axes from the PCA of chemical characters in the studied species of *Crataegus* genus.

Eigenvectors	Component		
1	2	3
(F) Total phenol	0.22	−0.09	**0.27ᵃ**
(F) Total flavonoid	0.23	−0.09	0.12
(F) Antioxidant capacity	0.22	0.06	**0.25ᵃ**
(F) Total anthocyanin	0.18	**−0.24ᵃ**	**0.27ᵃ**
(F) Ortho-diphenol	0.23	−0.11	0.02
(F) Hydroxycinnamic acid	**0.24ᵃ**	−0.08	−0.03
(F) Flavonol	0.23	−0.14	−0.08
(F) Chlorogenic acid	0.16	**0.34ᵃ**	−0.08
(F) Epicatechin	**0.24ᵃ**	0.10	−0.05
(F) *p*-coumaric acid	−0.06	0.21	**0.57ᵃ**
(F) Procyanidin	0.21	−0.21	0.06
(F) Ferulic acid	0.01	**0.46ᵃ**	0.06
(F) Rutin	**0.24ᵃ**	0.06	0.09
(F) Quercetin-3-O-glucoside	0.22	−0.18	−0.08
(F) Cinnamic acid	0.20	**0.24ᵃ**	−0.02
(F) Kaempferol-3-O-glucoside	0.18	**−0.24ᵃ**	**−0.27ᵃ**
(F) Apigenin	−0.20	**−0.25ᵃ**	−0.03
(L) Total phenol	0.21	−0.22	−0.03
(L) Total flavonoid	**0.24ᵃ**	−0.03	0.08
(L) Antioxidant capacity	0.15	**0.36ᵃ**	−0.04
(L) Total anthocyanin	0.22	0.17	−0.06
(L) Ortho-diphenol	**0.24ᵃ**	0.10	0.05
(L) Hydroxycinnamic acid	**0.24ᵃ**	0.04	−0.12
(L) Flavonol	−0.10	−0.17	**0.56ᵃ**
*Eigenvalue*	*17.04*	*4.78*	*2.18*
*Components degree of significance*	**	**	**
*Variance (%)*	*71.00*	*19.91*	*9.09*
*∑ variance (%)*	*71.00*	*90.91*	*100.00*

**ᵃ**Bold values indicate the characteristics that most influence each PC (Eigenvalues are significant ≥ 0.24). Component degree of significance: *p < 0.05, **p < 0.01. *(F)* Fruit, *(L)* Leaf.

PC2 explained 19.91% of the total variance, with major contributions from fruit ferulic acid (0.46), leaf antioxidant capacity (0.36), fruit chlorogenic acid (0.34), fruit cinnamic acid (0.24), fruit total anthocyanin (−0.24), fruit kaempferol-3-O-glucoside (−0.24), and fruit apigenin (−0.25). The presence of antioxidant capacity and key phenolic acids as significant contributors to PC2 suggests that this component may capture variations in antioxidant potential and phenolic composition. The negative loadings of total anthocyanin and kaempferol-3-O-glucoside indicate an inverse relationship with other traits in this component, implying possible trade-offs between different classes of phenolic compounds.

PC3, explaining 9.09% of the total variance, was primarily associated with fruit *p*-coumaric acid (0.57), leaf flavonol (0.56), fruit total phenol (0.27), fruit total anthocyanin (0.27), fruit antioxidant capacity (0.25), and fruit kaempferol-3-O-glucoside (−0.27). These results suggest that PC3 captures additional variation in specific flavonoid and phenolic acid contents, further reinforcing the role of these compounds in determining the chemical profile of *Crataegus* species.

The cumulative variance explained by the first three principal components demonstrates that these axes sufficiently represent the chemical diversity among the studied species. The statistical significance of the components (PC1, PC2, and PC3, *p* < 0.01) indicates that these principal components are robust in capturing meaningful variation in the dataset. These findings suggest that the phenolic composition and antioxidant-related traits serve as key differentiating factors among the studied *Crataegus* species and could be used as reliable markers for genetic and chemotaxonomic characterization.

Our PCA results show partial similarity with the findings of similar studies conducted in Iran [1] and Türkiye [7]. These differences may arise due to variations in environmental conditions, genetic factors, or methodological approaches between the studies. Such discrepancies highlight the potential influence of local ecological factors or specific experimental designs on the outcomes, suggesting that while general trends may be observed, regional differences should also be considered when interpreting the results.

**Fig 2:**
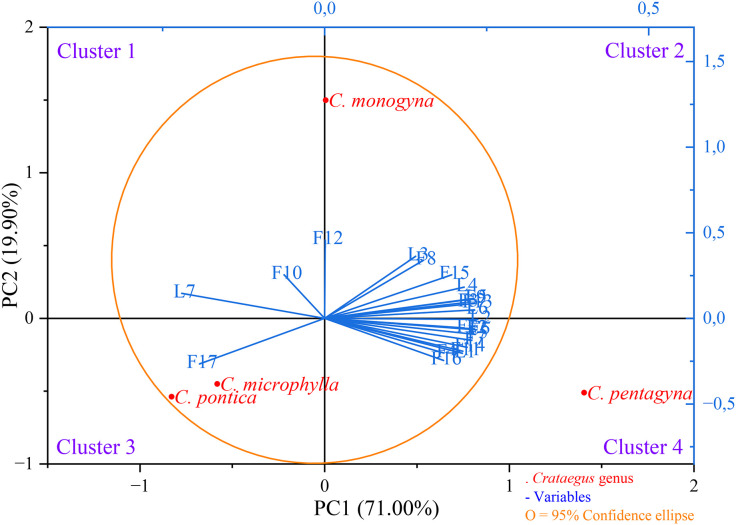
Biplot for the studied species of *Crataegus* genus based on PC1/PC2 of chemical traits. For abbreviations, please see Table 1.

Biplot for the four studied species of *Crataegus* genus based on PC1/PC2 of chemical traits is shown in Fig 2. Thus, the first two components (PC1 = 71.00% and PC2 = 19.91%) account for 90.91% of the total variation. The exploratory PCA score plot is presented in S1 Fig.

The biplot analysis reveals a distinct distribution pattern, with *Crataegus* species clustering into three regions, while the analyzed variables are dispersed across four regions. This differentiation suggests that the chemical traits exhibit more variation than the species themselves, potentially indicating species-specific metabolic adaptations or environmental influences.

In Cluster 1, p-coumaric acid in fruits and flavonol in leaves were grouped, implying a potential co-regulation of these compounds in certain *Crataegus* species. The presence of these metabolites in the same cluster suggests a biochemical link, possibly related to shared biosynthetic pathways or similar ecological functions.

Cluster 2 was characterized by *C. monogyna* and a set of fruit and leaf traits, including fruit antioxidant capacity, chlorogenic acid, epicatechin, ferulic acid, rutin, cinnamic acid, as well as leaf antioxidant capacity, total anthocyanin, ortho-diphenol, and hydroxycinnamic acid. The clustering of *C. monogyna* with multiple phenolic compounds and antioxidant-related traits indicates that this species may possess a distinct metabolic profile, potentially contributing to higher antioxidant capacity. The presence of hydroxycinnamic acids and flavonoids in both fruit and leaves suggests a coordinated defense mechanism against oxidative stress, which may be an adaptive advantage in specific ecological conditions.

Cluster 3 included *C. microphylla* and *C. pontica* along with apigenin. The association between these species and a single metabolite suggests a species-specific accumulation of apigenin, possibly linked to unique physiological or ecological functions. Apigenin is known for its antioxidant and anti-inflammatory properties, and its clustering with these species may indicate a role in stress response or environmental adaptation.

Cluster 4 encompassed *C. pentagyna* and a broad range of metabolites, including total phenol, total flavonoid, total anthocyanin, ortho-diphenol, hydroxycinnamic acid, flavonol, procyanidin, quercetin-3-O-glucoside, and kaempferol-3-O-glucoside in fruits, as well as total phenol and total flavonoid in leaves. The diverse metabolite profile of ‘*C. pentagyna*’ suggests a rich and complex secondary metabolism, potentially offering strong antioxidative and protective properties. The accumulation of multiple flavonoids and phenolic compounds in both fruits and leaves highlights the potential functional significance of this species, possibly reflecting an adaptation to environmental stressors or enhanced bioactivity.

Overall, the observed clustering patterns suggest that different *Crataegus* species exhibit distinct chemical profiles, with certain species accumulating specific phenolic compounds more prominently. The variation in phenolic composition across species and plant parts emphasizes the role of both genetic and environmental factors in metabolite accumulation.

The fact that *Crataegus pentagyna* falls outside the 95% confidence ellipse indicates that this species exhibits a distinct chemical composition compared with the other studied *Crataegus* species [47]. This differentiation can be attributed to several possible factors.

Firstly, the phenolic profile and metabolic composition of *C. pentagyna* differ significantly from those of the other species. This variation may result from the species’ genetic makeup or be influenced by the ecological conditions in which it grows. Differences in soil composition, microclimatic conditions, and environmental stress factors likely play a crucial role in shaping its metabolite composition.

Secondly, the metabolic pathways of *C. pentagyna* may differ from those of other *Crataegus* species, particularly in terms of the production or accumulation of specific phenolic compounds. The notably high levels of total phenol, total flavonoid, total anthocyanin, ortho-diphenol, hydroxycinnamic acid, flavonol, procyanidin, quercetin-3-O-glucoside, and kaempferol-3-O-glucoside in the fruit suggest that this species possesses a unique chemical profile.

Moreover, the fact that *C. pentagyna* lies outside the 95% confidence ellipse suggests a high degree of metabolic diversity, implying the presence of distinct defense mechanisms or adaptive strategies. Considering the protective role of phenolic compounds against environmental stresses, it is likely that *C. pentagyna* has developed a unique chemical response to specific ecological conditions or abiotic stress factors.

In conclusion, the statistical separation of *C. pentagyna* from the other species underscores the need for further investigation into its biochemical and physiological characteristics. A deeper understanding of phenolic diversity and chemical composition differences among species will contribute to elucidating interspecific variations at both genetic and ecological levels, as well as their potential functional implications in terms of bioavailability and health-related benefits.

Considering the limited number of sampled materials included in the present dataset, the PCA results should be interpreted primarily as exploratory visualization patterns rather than definitive evidence of biological differentiation.

### 3.5. Heat map analysis (HMA)

**Fig 3:**
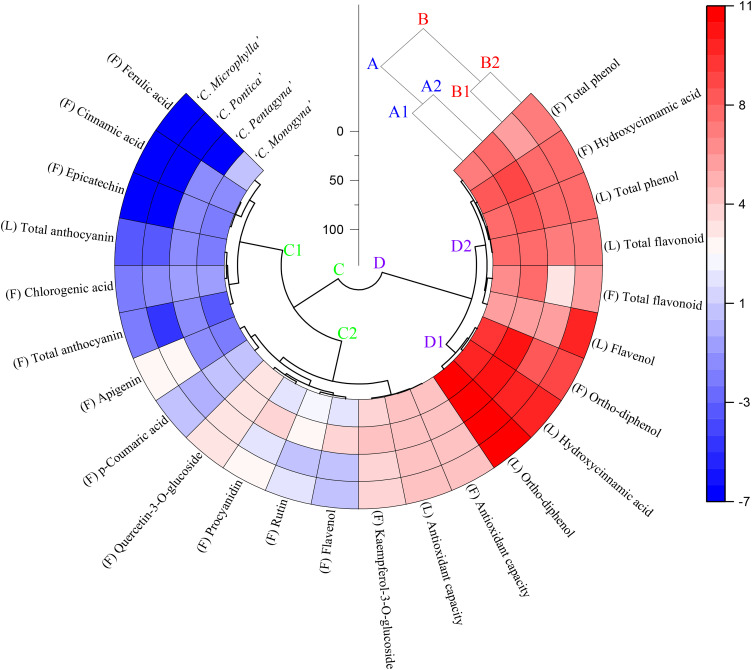
Visualization of clustering patterns of the studied species of *Crataegus* genus and variables based on chemical characterizations using a heat map. For abbreviations, please see Table 1. *(F)* Fruit, *(L)* Leaf.

Visualization of clustering patterns of four species of *Crataegus* genus and variables based on chemical characterizations using a heat map is shown in Fig 3. The species are categorized into two main groups, A and B, which are further subdivided into A1, A2, B1, and B2, representing distinct *Crataegus* species: *C. monogyna*, *C. pentagyna*, *C. pontica*, and *C. microphylla*.

Similarly, the chemical content is divided into two main groups, C and D, with each group further split into two subgroups (C1, C2, D1, and D2). This subdivision appears to correspond to the specific types of compounds found in the fruits and leaves of the *Crataegus* species. Group C1 includes compounds such as ferulic acid, cinnamic acid, epicatechin, chlorogenic acid, and total anthocyanin in both fruits and leaves, indicating the presence of antioxidants and flavonoids that are important for the health benefits of these plants. Group C2 contains additional compounds, including apigenin, *p*-coumaric acid, quercetin-3-O-glucoside, procyanidin, and rutin, all of which are known for their antioxidant properties, and their variation between fruit and leaf tissues highlights the potential for tissue-specific bioactivity.

Groups D1 and D2 represent different chemical compounds with potential pharmacological implications. D1 is characterized by the presence of ortho-diphenol and hydroxycinnamic acid in the leaves and fruits, suggesting their role in antioxidant and anti-inflammatory properties. D2 encompasses flavonoids, phenolic acids, and other bioactive compounds such as total flavonoids and total phenols, particularly in the fruit, which are linked to antioxidant and anti-carcinogenic effects.

The use of a clear classification system for both species and chemical components enhances the understanding of the chemical diversity within *Crataegus* species. It allows for more targeted research into the specific chemical constituents that may have therapeutic potential. For example, the higher antioxidant content in the fruits of certain species could be a focal point for exploring their role in human health, particularly concerning diseases that benefit from antioxidant-rich diets.

In addition, the precise categorization of chemical compounds and their variations between species and plant parts (fruits vs. leaves) offers valuable insight into the ecological and biochemical diversity of *Crataegus* species. This could inform agricultural practices, such as which species or plant parts are most beneficial for specific health-related uses or industrial applications.

Heat map analysis, which has been successfully utilized in the breeding studies of various fruit species, provides valuable insights into patterns of variation and underlying relationships among genotypes [48–51]. The heat map analysis results are partially consistent with the findings of similar studies conducted in Iran [34] and Türkiye [7]. This alignment suggests that the observed patterns may reflect common underlying trends across different geographic regions. However, the partial discrepancy between the studies could indicate the influence of region-specific factors, such as climate, soil conditions, or genetic diversity, which may contribute to the observed variations. These findings highlight the importance of considering both global trends and local environmental conditions when interpreting data from different regions.

In conclusion, the hierarchical and systematic classification of both the *Crataegus* species and their chemical profiles offers a comprehensive framework for understanding their potential in terms of chemical diversity and therapeutic applications. Further studies should focus on the biological activity of these compounds, as well as the potential for developing *Crataegus*-based functional foods or medicinal products. This approach could lead to advancements in both pharmacological research and the development of sustainable agricultural practices.

## 4. Conclusions

The studied *Crataegus* species exhibited considerable variation in phenolic compounds and antioxidant-related traits, indicating substantial phytochemical diversity among the evaluated materials. Among the species, *C. pentagyna* generally showed higher levels of several phenolic-related compounds and antioxidant capacity, suggesting its strong phytochemical potential. Correlation, PCA, HMA, and MRA analyses revealed significant associations among phenolic compounds and antioxidant-related variables, indicating coordinated metabolic relationships within the analyzed dataset. The findings also suggest that both genetic background and ecological conditions may influence phenolic accumulation and biochemical composition in *Crataegus* species. Overall, the results demonstrate that *Crataegus* species represent valuable sources of bioactive compounds with potential applications in functional foods, nutraceuticals, and plant-based products. Further studies integrating molecular and metabolomic approaches are needed to better understand the regulatory mechanisms underlying phenolic biosynthesis in these species.

## Supporting information

S1 File Raw data of biochemical properties of fruit and leaf in the studied germplasm of *Crataegus.* (XLSX)

S2 File Inclusivity-in-global-research-questionnaire. (DOCX)

S1 Fig The exploratory PCA score plot. (DOCX)
