# Supplementary material for: Biochemical characterizations of leaves and fruits in Crataegus monogyna Jacq., C. pontica K.Koch, C. microphylla K.Koch, and C. pentagyna Waldst. & Kit. ex Willd
Source: PLoS One. 2026 Jul 6;21(7):e0352757. doi: 10.1371/journal.pone.0352757 (PMC13336193; doi:10.1371/journal.pone.0352757)
Supplement: S1 Fig — (DOCX) [file pone.0352757.s003.docx]

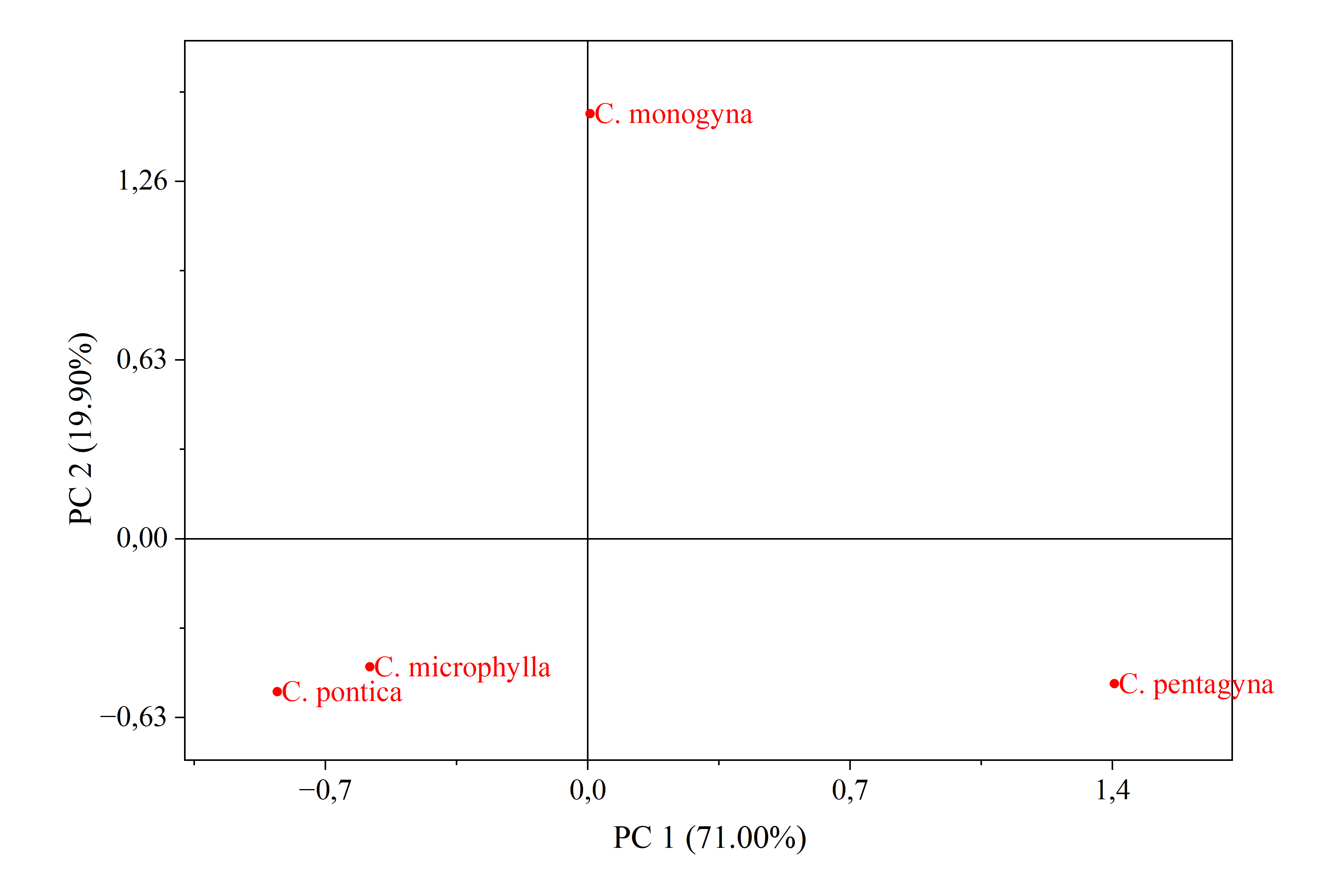


**S1 Fig.** Exploratory PCA score plot of the analyzed *Crataegus* samples based on biochemical traits.
