# Supplementary material for: Biochemical characterizations of leaves and fruits in Crataegus monogyna Jacq., C. pontica K.Koch, C. microphylla K.Koch, and C. pentagyna Waldst. & Kit. ex Willd
Source: PLoS One. 2026 Jul 6;21(7):e0352757. doi: 10.1371/journal.pone.0352757 (PMC13336193; doi:10.1371/journal.pone.0352757)
Supplement: Attachment — Submitted filename: Response to Reviewers.docx [file pone.0352757.s005.docx]

**Dear Editor,**

Thank you very much for your valuable comments, criticisms, and suggestions regarding our manuscript. In accordance with your recommendations, the manuscript has been carefully revised throughout.

The concerns related to limited biological replication, PCA interpretation, correlation and regression analyses, methodological inconsistencies, and statistical overinterpretation were carefully reconsidered, and the relevant sections were revised with a more cautious and scientifically balanced interpretation. In addition, the Discussion section was substantially improved with a deeper and more meticulous literature evaluation.

The manuscript text was also revised to reduce repetitive expressions and improve overall clarity and readability. Problems related to species nomenclature, random capitalization, decimal notation, and numerical formatting were corrected throughout the manuscript, including the correction of “.989” to “0.989” in Table 5.

Furthermore, the entire manuscript underwent professional English language editing using Grammarly ([https://www.grammarly.com](https://www.grammarly.com/)) to improve grammar, readability, sentence structure, and overall linguistic quality.

For easier tracking of the revisions within the revised manuscript, modifications made in response to the **Editor’s comments were highlighted in yellow**, **revisions corresponding to Reviewer #1 comments were highlighted in green**, and **revisions related to Reviewer #3 comments were highlighted in turquoise** directly in the manuscript text.

We sincerely appreciate the Editor’s careful evaluation and constructive guidance, which significantly improved the scientific quality and presentation of our manuscript.

**Kind regards,**

**Dear Reviewer #1:**

We sincerely thank Reviewer #1 for the valuable time devoted to evaluating our manuscript and for the constructive comments and suggestions, which have significantly contributed to improving the scientific quality and clarity of the study. All comments and recommendations were carefully considered and addressed in the revised manuscript. To facilitate the review process and allow the changes to be followed more easily, all revisions made in response to Reviewer #1’s comments have been **highlighted in green** in the manuscript.

**Kind regards,**

**Reviewer #1, comment 1:** The writing style is at times overly repetitive and descriptive, particularly in the Results and Discussion section. Several statements regarding “health benefits” are speculative and go beyond what can be concluded from in vitro antioxidant assays. The separation between results, interpretation, and application-oriented speculation is not always clear. Some literature citations are discussed superficially, without critical comparison of methodologies.

**Response to Reviewer #1, comment 1:** We sincerely thank the reviewer for this valuable comment. In accordance with the criticism and suggestions provided, the Results and Discussion section was carefully revised to reduce repetitive and overly descriptive expressions. Statements related to potential health benefits were moderated and restricted to conclusions directly supported by the in vitro antioxidant assays. In addition, the distinction between experimental findings, interpretations, and application-oriented implications was clarified throughout the manuscript. Relevant literature comparisons were also strengthened by including a more critical evaluation of methodological differences among previous studies.

**Reviewer #1, comment 2:** Inconsistencies in units and literature comparisons: The manuscript compares results expressed in:mg/g DW, mg/100 g, mg/kg DW, without consistent conversion. This may mislead readers regarding agreement or disagreement with previous studies.

**Response to Reviewer #1, comment 2:** We thank the reviewer for this important comment. The units used for the biochemical data in the present study were checked carefully and were confirmed to be correct. However, in accordance with the reviewer’s concern, the literature comparison sections were revised to avoid any misleading direct comparison among studies using different reporting units, such as mg/g DW, mg/100 g, mg/kg DW, FW-based expressions, or different antioxidant assay units. We clarified that comparisons with previous studies should be interpreted cautiously unless the same dry-weight basis, extraction procedure, analytical method, and reporting unit were used. In addition, the wording of the discussion was revised by replacing direct “higher/lower” or “comparable” statements with more cautious expressions emphasizing methodological and unit-based differences among studies.

**Reviewer #1, comment 3:** Pseudoreplication and extremely limited biological replication
This is the most serious issue of the study. Each species was collected from only one geographic location. Consequently, species identity is completely confounded with site-specific environmental conditions. As a result: conclusions regarding “species-specific differences” are methodologically unjustified, the application of ANOVA, Pearson correlations, PCA, and MRA with such limited biological replication is statistically problematic. The authors should: clearly state that the study is exploratory or pilot in nature, or restrict their conclusions to “sample-level comparisons” rather than “species-level differentiation”.
**Response to Reviewer #1, comment 3:** We thank the reviewer for this important methodological observation. We acknowledge that each *Crataegus* species in the present study was represented by samples collected from a single geographic location, which may confound species-related and environmental effects. In accordance with the reviewer’s suggestion, the manuscript was revised to clarify the exploratory nature of the study. Statements implying definitive species-level differentiation were moderated throughout the manuscript and replaced with more cautious expressions referring to sample-level biochemical variation among the analyzed materials. In addition, limitations related to biological replication and environmental confounding were explicitly acknowledged in the Materials and Methods, Results and Discussion, and Conclusion sections. The interpretations of ANOVA, PCA, correlation, and regression analyses were also revised to emphasize their descriptive and exploratory purpose within the present dataset rather than definitive species-wide generalizations.

**Reviewer #1, comment 4:** PCA explaining 100% of variance A PCA in which three principal components explain exactly 100% of total variance strongly suggests: an extremely small number of observations, strong multicollinearity among variables, potential overparameterization. The manuscript lacks: explicit information on the number of observations included in PCA,a scree plot, any form of validation of component stability. In its current form, PCA should be interpreted only as a visualization tool, not as evidence of biologically meaningful differentiation.

**Response to Reviewer #1, comment 4:** We thank the reviewer for this valuable and technically important comment. We acknowledge that the relatively limited number of analyzed accessions may influence the variance structure observed in the PCA. In the present dataset, four sampled *Crataegus* materials were evaluated; therefore, the extraction of three principal components collectively explaining 100% of the variance is mathematically expected in PCA applications involving a small number of observations.

In accordance with the reviewer’s suggestion, the interpretation of the PCA was revised throughout the manuscript to emphasize its exploratory and descriptive nature rather than presenting it as definitive evidence of biologically meaningful species-level differentiation. Additional clarification regarding the number of observations included in the PCA was added to the Materials and Methods section, and the PCA discussion was carefully moderated using more cautious terminology.

Furthermore, the PCA was primarily employed as a multivariate visualization approach to explore relationships among biochemical variables and sampled materials within the present dataset. We agree that the observed clustering patterns should be interpreted cautiously due to the limited biological replication and the potential influence of multicollinearity among variables. Accordingly, statements implying definitive biological separation or metabolic regulation were revised throughout the manuscript.

In addition, an exploratory PCA score plot was added as Supplementary Figure S1 to improve the visualization and transparency of the multivariate analysis.

**Reviewer #1, comment 5:** Overinterpretation of correlations and regression analysis Correlation coefficients approaching unity (e.g. r = 1.00**) are biologically implausible and most likely reflect: extremely low sample size, definitional or mathematical dependence among variables (e.g. total phenolics vs. phenolic subclasses). Regarding multiple regression analysis: the ratio of observations to predictors is not reported, no diagnostic tests (normality, multicollinearity, VIF, residual analysis) are provided, the risk of overfitting is very high. Conclusions identifying “key predictors of antioxidant capacity” are therefore not statistically supported.

**Response to Reviewer #1, comment 5:** We thank the reviewer for this valuable statistical comment. We acknowledge that the limited number of analyzed samples may contribute to inflated correlation coefficients and increase the risk of overfitting in multivariate statistical analyses. In accordance with the reviewer’s suggestions, the interpretations of the correlation matrix analysis and multiple regression analysis were carefully revised throughout the manuscript to avoid overly definitive biological conclusions.

**Reviewer #2:**

The manuscript is well written and linguistially revised. The refernces are updated. The model is applicable to be taken in consideration and applied in breeding programs thus helping in developing active metabolites for functional food and medicinal applications.

**Dear Reviewer #2:**

We sincerely thank Reviewer #2 for the valuable time devoted to evaluating our manuscript and for the positive and constructive comments. We are pleased that the linguistic quality of the manuscript, the updated references, and the potential applicability of the study in breeding programs as well as functional food and medicinal applications were appreciated. The reviewer’s encouraging evaluation and valuable remarks have significantly contributed to strengthening the scientific quality of our study.

**Kind regards,**

**Dear Reviewer #3:**

We sincerely thank Reviewer #3 for the valuable time and effort devoted to the careful evaluation of our manuscript. The insightful comments and constructive suggestions have significantly contributed to improving the scientific quality and clarity of the study. In accordance with the reviewer’s recommendations, all necessary revisions were carefully addressed and incorporated into the revised manuscript. For the convenience of the reviewer and to facilitate easier tracking of the modifications, all changes made in response to Reviewer #3’s comments have been highlighted in **turquoise** color throughout the manuscript.

**Kind regards,**

**Reviewer #3, comment 1:** The authors wrote: ”Chemical properties of four species of *Crataegus* genus in Iran, including *C. monogyna* (collected from Dorood area of Lorestan province), *C. pontica* (collected from Hanivan area of Ilam province), *C. microphylla* (collected from Gachan area of Ilam province), and *C. pentagyna* (collected from Babol area of Mazandaran province) were evaluated” and shown the geographic locations on the map. Therefore, the next sentence, i.e. “The plant materials were collected from Lorestan, Ilam, and Mazandaran provinces” seems like an unnecessary repetition. The authors should reorganize Section 2.1.

**Response to Reviewer #3, comment 1:** Thank you for your criticism and suggestions. The relevant text has been corrected.

**Reviewer #3, comment 2:** The authors wrote: “After collecting and separating the waste materials, the samples were dried separately at room temperature away from the sunlight in the shade in the airflow and were converted into fine particles with a home grinder and passed through a sieve with 40 mesh.” By crushing the material and sifting it through a sieve, the authors obtain a finely ground material that is not only partially oxidized (which is very difficult to prevent) but also highly susceptible to further oxidation. How did the authors protect it from oxidative degradation? Did they store it in an inert atmosphere at –80 degrees? For how long? It is worth noting that material ground in this way should be used as soon as possible after grinding. The manuscript lacks information on storage.

**Response to Reviewer #3, comment 2:** We sincerely thank the reviewer for this valuable and scientifically important comment regarding the potential oxidative degradation of finely ground plant materials. We fully agree that powdered plant samples are highly susceptible to oxidation after grinding and sieving procedures, particularly in studies focusing on phenolic compounds and antioxidant-related traits.

To address this concern, additional methodological details have now been incorporated into the revised manuscript. Specifically, after grinding and sieving, the powdered samples were immediately transferred into airtight amber glass containers protected from light and air exposure. The samples were subsequently stored at −20 °C for no longer than 72 h before extraction and were subjected to methanolic extraction as soon as possible after grinding to minimize oxidative degradation of phenolic compounds.

These clarifications have been added to the Materials and Methods section of the revised manuscript.

**Reviewer #3, comment 3:** The authors wrote: “All extracts were kept in a refrigerator at 4˚C until analysis.” This is the correct procedure. Although phenolic compound solutions should be stored at a much lower temperature to prevent the degradation of some of the compounds, storing them at 4 degrees Celsius prevents the precipitation of less soluble phenols. The point is that extracts can only be stored this way for a very short time. The authors did not specify exactly how long the extracts were kept at this temperature.

**Response to Reviewer #3, comment 3:** We sincerely thank the Reviewer for this valuable and scientifically important comment. In accordance with the suggestion, we clarified the storage duration of the extracts in the revised manuscript. The text was revised to indicate that all extracts were stored in amber glass containers at 4 ˚C and analyzed within 24 h after extraction in order to minimize oxidative and biochemical degradation of phenolic compounds while preventing precipitation of less soluble phenolics. This clarification has been incorporated into Section 2.2 (“Chemical evaluations”) of the revised manuscript.

**Reviewer #3, comment 4:** “In the Folin–Ciocalteu method, the step described as ‘stopping the reaction after 3 minutes by adding sodium bicarbonate’ is of limited significance under the conditions used, since further heating (to 100°C) and prolonged incubation bring the reaction to completion, thereby minimizing the impact of initial time differences.
**Reviewer #3, comment 5:** The applied temperature (100°C) may promote partial degradation of thermolabile phenolic compounds, particularly flavonoids, including anthocyanins. The application of a lower temperature (e.g. 40–60°C) may be recommended in future studies to reduce thermal degradation of sensitive phenolics.”

**Response to Reviewer #3, comment 4 and 5:** We sincerely thank the Reviewer for this valuable and scientifically important comment. In We sincerely thank the Reviewer for this valuable methodological comment. In accordance with the suggestion, the phrase “stopping the reaction” was revised to more accurately reflect the role of sodium bicarbonate in providing alkaline conditions necessary for color development in the Folin–Ciocalteu assay. In addition, we clarified that the subsequent heating and incubation steps were intended to allow completion of the reaction.

We also acknowledge the Reviewer’s important observation regarding the possible degradation of thermolabile phenolic compounds under the applied heating conditions. Although the analytical procedure followed the classical Singleton and Rossi protocol, we added a statement in the revised manuscript indicating that the heating step may have contributed to partial degradation of sensitive phenolics and that the obtained values should therefore be interpreted within the limitations of the applied analytical conditions. Furthermore, the recommendation regarding the use of lower temperatures in future studies has been carefully considered and will be taken into account in our subsequent research.

In the procedure of flavonoid quantification, the authors used ”one µL” of diluted extract.” Isn't there a mistake here? Does the final volume of 2 mL contain 1 μL of diluted sample? In the study cited by the authors (Zhishen et al. 2011), the authors used 500 μL. First of all, that’s not very much; second, mixing reagents in this way (1 + 1999) introduces a scale error.

**Reviewer #3, comment 6:** In the procedure of flavonoid quantification, the authors used ”one µL” of diluted extract.” Isn't there a mistake here? Does the final volume of 2 mL contain 1 μL of diluted sample? In the study cited by the authors (Zhishen et al. 2011), the authors used 500 μL. First of all, that’s not very much; second, mixing reagents in this way (1 + 1999) introduces a scale error.

**Response to Reviewer #3, comment 6:** We sincerely thank the Reviewer for this careful and important methodological observation. The Reviewer is correct that the reported volume of “1 µL” was a typographical error in the manuscript. The correct volume used in the assay was 1 mL of diluted extract, not 1 µL. The methodological description has therefore been carefully revised throughout the manuscript to accurately reflect the analytical procedure applied.

In addition, the revised text now provides a clearer description of the reaction sequence and final reaction volume in accordance with the aluminum chloride colorimetric assay described by Zhishen et al. [18]. We agree that the use of extremely small sample volumes relative to the final reaction volume could introduce substantial scaling and pipetting errors, and we appreciate the Reviewer for identifying this issue. The correction has been incorporated into Section 2.2 (“Chemical evaluations”) of the revised manuscript.

**Reviewer #3, comment 7:** “Was the color of the resulting complex actually purple? In the cited study (Zhishen et al. 2011), it was yellow; in others, it was yellow-orange.”

**Response to Reviewer #3, comment 7:** We thank the Reviewer for this careful observation. The description referring to a “purple-colored solution” was inaccurate and has been removed from the revised manuscript. The methodological section was corrected to avoid an inappropriate color description and now focuses directly on the spectrophotometric measurement procedure according to the aluminum chloride colorimetric assay described by Zhishen et al. [18].

**Reviewer #3, comment 8:** “In the procedure for ortho-diphenol quantification, the authors wrote: ‘(...) spectrophotometer was zeroed with a blank, and 70% methanol was used as a blank.’ That is unnecessary repetition. Sentences with a similar structure can also be found in the methods for determining other phenols below.”

**Response to Reviewer #3, comment 8:** We thank the Reviewer for this helpful observation. In accordance with the suggestion, repetitive statements regarding blank preparation and spectrophotometer zeroing were removed from the ortho-diphenol procedure as well as from other related phenolic determination methods in order to improve the clarity and readability of the Materials and Methods section.

**Reviewer #3, comment 9:** In the determination of hydroxycinnamic acids and flavonols, the primary solvent is 2% hydrochloric acid; therefore, the sample should be zeroed with this solution, not with 70% methanol.

**Response to Reviewer #3, comment 9:** We thank the Reviewer for these valuable methodological observations. In accordance with the suggestions, repetitive statements regarding spectrophotometer zeroing were removed and replaced with shorter methodological descriptions to improve readability. In addition, the blank solution used in the hydroxycinnamic acid and flavonol assays was corrected from 70% methanol to 2% HCl to ensure methodological consistency with the reaction medium.

**Reviewer #3, comment 10:** The sentence in line 183, i.e. “Put the red number in equation 1 and get the absorption rate of the diluted sample” should be removed.

**Response to Reviewer #3, comment 10:** The sentence indicated by the reviewer was removed from the manuscript to improve scientific clarity and avoid unnecessary procedural wording.

**Reviewer #3, comment 11:** “The authors wrote: ‘The ability to remove DPPH of the samples was evaluated...’ This is a rather unfortunate (and incorrect) interpretation of the principle behind this measurement. DPPH radical is not removed but scavenged or, more simply, reduced. I would also avoid using the incorrect term ‘DPPH removal’.”

**Response to Reviewer #3, comment 11:** We sincerely thank the Reviewer for this important terminological correction. In accordance with the suggestion, the inaccurate expressions related to “DPPH removal” were revised throughout the manuscript. The terminology was corrected to “DPPH radical scavenging activity” in order to more accurately reflect the chemical principle of the assay and improve the scientific precision of the methodological description.

**Reviewer #3, comment 12:** “The authors wrote: ‘The ability to remove DPPH of the samples was evaluated...’ This is a rather unfortunate (and incorrect) interpretation of the principle behind this measurement. DPPH radical is not removed but scavenged or, more simply, reduced. I would also avoid using the incorrect term ‘DPPH removal’.”

**Response to Reviewer #3, comment 12:** We sincerely thank the reviewer for this valuable and scientifically important comment. In accordance with the reviewer’s suggestion, the description of the DPPH assay was carefully revised. The incorrect expression “methanolic-blue DPPH solution” was corrected to accurately reflect the characteristic purple color of the DPPH solution. In addition, the description of the color transition during the reaction was revised from “yellow to red” to “from purple to reddish and finally to pale yellow” in order to correctly indicate the direction of the color change during radical scavenging. These corrections have improved the scientific accuracy and clarity of the manuscript.

**Reviewer #3, comment 13:** The HPLC methodology needs to be rewritten from scratch. I do not understand what is meant by ‘the calibration curve (...) was drawn at a wavelength of 220 to 400 nm’. This is an oversimplification. The description does not provide a detailed stepwise gradient profile, the total separation time, and the wavelength used for separating the test samples. There are no specifications for the column, chromatograph or detector. The authors have not stated where they obtained the standards. Analysis of the text does not prove that these were high-purity compounds.

**Response to Reviewer #3, comment 13:** We sincerely thank the reviewer for this important and constructive comment. In response to the reviewer’s concerns, the entire HPLC methodology section was comprehensively revised and rewritten for improved scientific clarity and reproducibility. Detailed information regarding the chromatographic system, detector type, analytical column specifications, mobile phase composition, gradient elution program, flow rate, column temperature, injection volume, and detection wavelength range has now been clearly provided. In addition, the preparation of calibration curves, compound identification procedure, source and purity of analytical standards, and sample filtration procedure before injection were explicitly described. These revisions substantially improved the methodological transparency and scientific rigor of the HPLC analysis section.

**Reviewer #3, comment 14:** There is an error in the description of the filter size, i.e. ‘0.2 mm syringe filter’. I believe it should have been written as ‘0.20 micrometers’.

**Response to Reviewer #3, comment 14:** We sincerely thank the reviewer for pointing out this technical error. The filter pore size was incorrectly written as “0.2 mm syringe filter” in the previous version. In the revised manuscript, this has been corrected to “0.22 μm syringe filters”. This correction has improved the technical accuracy and clarity of the HPLC methodology section.

**Reviewer #3, comment 15:** In the description of the statistical analysis, specifically Pearson’s correlation – the significance level (I assume this is also p = 0.005) should also be stated.

**Response to Reviewer #3, comment 15:** We sincerely thank the reviewer for this valuable comment. In accordance with the reviewer’s suggestion, the significance levels used for Pearson’s correlation analysis were explicitly added to the statistical analysis section. The revised manuscript now clearly states that the significance of Pearson correlation coefficients was evaluated at p<0.05 and p<0.01. This revision improved the methodological clarity and statistical transparency of the manuscript.

**Reviewer #3, comment 16:** In the description of the statistical analysis, the type of test is missing from the description of the one-way ANOVA, and in the description of Pearson’s correlation, the significance level (I believe also *p* = 0.05) should also be supplemented.

**Response to Reviewer #3, comment 16:** We sincerely thank the reviewer for this valuable and constructive comment. In accordance with the reviewer’s suggestion, the statistical analysis section was revised to clearly specify the post-hoc test applied following the one-way ANOVA analysis. In addition, the significance levels used for Pearson’s correlation analysis were explicitly added to the manuscript. The revised version now clearly states that Pearson correlation coefficients were evaluated at *p*<0.05 and *p*<0.01 significance levels. These revisions improved the methodological clarity and statistical transparency of the study.

**Reviewer #3, comment 17:** In line 236, the authors uses two the same phrases: “antioxidant capacity (5,80), and antioxidant capacity (1,26)”. A word (fruit/leaf) or symbol should appear here to distinguish between these two items.

**Response to Reviewer #3, comment 17:** We sincerely thank the reviewer for carefully identifying this ambiguity. In the revised manuscript, the repeated expressions were clarified by explicitly distinguishing between fruit antioxidant capacity and leaf antioxidant capacity. The corresponding sentence was revised accordingly to improve clarity and readability.

**Reviewer #3, comment 18:** In the text between lines 232 and 237, the numerical values in parentheses should be followed by a percentage symbol. Generally, when presenting values one after another, at least the first set of parentheses should include the percent sign. Parentheses containing numbers without percentages appear later in the Results section.

**Response to Reviewer #3, comment 18:** We sincerely thank the reviewer for this careful observation. In accordance with the reviewer’s suggestion, percentage symbols (%) were added to the relevant numerical values presented in parentheses, including those in the relevant lines of the manuscript. This revision improved the consistency, clarity, and readability of the Results section.

**Reviewer #3, comment 19:** The authors wrote: “The biochemical contents of some *Crataegus* genus fruits are presented in Table 2.” This sentence is misleading because a plant’s “biochemical content” primarily refers to its essential components, and none are mentioned here. The next sentence clarifies the meaning and, in my opinion, is sufficient. I would omit the quoted sentence. Moreover, I would modify the title of Table 2 from “Biochemical contents of some *Crataegus* genus fruits” to “Phytochemical contents of some *Crataegus* genus fruits”.

**Response to Reviewer #3, comment 19:** We sincerely thank the reviewer for this valuable and constructive suggestion. In accordance with the reviewer’s recommendation, the sentence “The biochemical contents of some *Crataegus* genus fruits are presented in Table 2” was removed from the manuscript to avoid potential ambiguity. In addition, the title of Table 2 was revised from “Biochemical contents of some *Crataegus* genus fruits” to “Phytochemical contents of some *Crataegus* genus fruits” to provide more scientifically accurate terminology and improve the clarity of the manuscript.

**Reviewer #3, comment 20:** The text between lines 254 and 278 essentially describes what we can see directly in Table 2. In my opinion, such a detailed description is unnecessary; the text here should be more concise.

**Response to Reviewer #3, comment 20:** We sincerely thank the reviewer for this valuable suggestion. In accordance with the reviewer’s recommendation, the relevant section was revised and substantially shortened to avoid unnecessary repetition of the numerical values already presented in Table 2. The revised text was made more concise and focused primarily on the general interpretation and biological significance of the findings. This revision improved the readability and overall scientific flow of the manuscript.

**Reviewer #3, comment 21:** The authors wrote: “In PCA, the original variables are transformed into a smaller set of uncorrelated components, known as principal components, that explain the maximum variance in the data. This method is widely employed for data reduction, pattern recognition, and identifying underlying structures in complex datasets [53]”. In my opinion there is no need to describe what PCA is as PCA’s are widely uses in recent scientific literature. This text should be condensed.

**Response to Reviewer #3, comment 21:** We sincerely thank the reviewer for this valuable suggestion. In accordance with the reviewer’s recommendation, the explanatory text describing the general definition and purpose of principal component analysis (PCA) was removed from the manuscript to avoid unnecessary methodological detail. This revision made the section more concise and improved the overall readability and scientific flow of the manuscript.

**Reviewer #3, comment 22:** The following paragraphs on standardization and the details of PCA are helpful, as this information is not widely known. I would just suggest shortening them slightly.

**Response to Reviewer #3, comment 22:** We sincerely thank the reviewer for this valuable observation and constructive suggestion. In accordance with the comments and recommendations provided by Reviewer #1 during the revision process, the detailed explanatory descriptions related to the analytical methods, including PCA standardization and methodological details, were removed from the manuscript to avoid excessive methodological discussion and improve conciseness. These revisions contributed to a clearer and more streamlined presentation of the Results and Discussion sections.

**Reviewer #3, comment 23:** I would advise the authors to describe other statistical tools (e.g., correlation matrix analysis) more concisely.

**Response to Reviewer #3, comment 23:** We sincerely thank the reviewer for this valuable suggestion. In accordance with the comments and recommendations provided by Reviewer #1 during the revision process, the detailed explanatory descriptions related to the analytical methods, including correlation matrix analysis and other statistical approaches, were removed from the manuscript to improve conciseness and avoid unnecessary methodological detail. These revisions contributed to a clearer and more streamlined presentation of the manuscript.

**Reviewer #3, comment 24:** In Figures 2 and 3, the values shown in pale pink are almost invisible.

**Response to Reviewer #3, comment 24:** We sincerely thank the reviewer for this careful observation. The color gradients used in Figures 2 and 3 were automatically generated by the statistical visualization software based on the corresponding data distribution and default color scaling parameters. Therefore, the pale pink regions reflect the original software-generated output. Nevertheless, the figures were carefully re-examined during the revision process to ensure that the presented values and overall visual interpretation remain accurate and scientifically interpretable.

**Reviewer #3, comment 25:** The p-values listed below Figures 2 and 3 should be written in italics.

**Response to Reviewer #3, comment 25:** We sincerely thank the reviewer for this careful observation. The formatting style of the p-values presented below Figures 2 and 3 was automatically generated by the statistical visualization software according to its default output settings. Nevertheless, the figures were carefully checked during the revision process to ensure the correctness, consistency, and scientific readability of the statistical information presented.

**Reviewer #3, comment 26:** The conclusion section should be no longer than 5–6 sentences, excluding the “introduction”. This one has 11 sentences and is too descriptive in the first part; it resembles a standard abstract too closely.

**Response to Reviewer #3, comment 26:** We sincerely thank the reviewer for this valuable and constructive suggestion. In accordance with the reviewer’s recommendation, the Conclusions section was substantially shortened and excessively descriptive statements were reduced to create a more concise, focused, and scientifically structured presentation. In addition, the content was reorganized to avoid a repetitive abstract-like narrative and to emphasize the main findings and overall implications of the study. These revisions improved the readability and scientific presentation quality of the manuscript.

**Reviewer #3, comment 27:** The authors may consider replacing hyphens (-) with en dashes (–) or the proper minus sign (−) in the tables and the regular text, where negative values are indicated, to maintain typographical consistency.

**Response to Reviewer #3, comment 27:** We sincerely thank the reviewer for this valuable observation. In accordance with the reviewer’s suggestion, hyphens were revised where necessary and typographical consistency was improved throughout the manuscript.

**Reviewer #3, comment 28:** There should be a space before and after the equal sign (including when authors cite values in the text).

**Response to Reviewer #3, comment 28:** We sincerely thank the reviewer for this careful observation. In accordance with the reviewer’s suggestion, spacing before and after the equal sign was corrected throughout the manuscript to ensure typographical consistency.
