# Supplementary material for: Biochemical characterizations of leaves and fruits in Crataegus monogyna Jacq., C. pontica K.Koch, C. microphylla K.Koch, and C. pentagyna Waldst. & Kit. ex Willd
Source: PLoS One. 2026 Jul 6;21(7):e0352757. doi: 10.1371/journal.pone.0352757 (PMC13336193; doi:10.1371/journal.pone.0352757)
Supplement: Attachment — Submitted filename: Responses to the Editor and Reviewer 3.docx [file pone.0352757.s006.docx]

**Dear Editor,**

We sincerely thank the Editor for the time, effort, and valuable comments devoted to the evaluation of our manuscript. The constructive suggestions and recommendations have significantly improved the scientific quality, clarity, and overall presentation of the manuscript. We have carefully addressed all comments and implemented the requested revisions throughout the manuscript. To facilitate the review process and enable easy identification of the modifications, all changes made in response to the Editor’s comments **have been highlighted in Yellow** in the revised manuscript.

**Editor Comment:** Fig. 2 and Fig. 3 still contain comma instead full stop to define decimal places. Please correct.

**Response:** Thank you for your careful observation. We have thoroughly revised the manuscript and corrected all decimal separators throughout the document. In addition, the former Figure 2 and Figure 3 have been reorganized and are now presented as **Table 5** and **Table 6**, respectively. All decimal values have been standardized using full stops (.) instead of commas (,), in accordance with the journal's formatting requirements. The revised tables have been carefully checked to ensure consistency and accuracy throughout the manuscript.

**Regards,**

**Dear Reviewer 3,**

We would like to express our sincere gratitude to Reviewer 3 for the careful evaluation of our manuscript and for the insightful and constructive comments provided. The reviewer’s suggestions have been extremely valuable in improving the methodological clarity, transparency, and scientific rigor of the study. We have carefully considered all comments and revised the manuscript accordingly. For the convenience of the reviewer, all modifications made in response to Reviewer 3’s comments **have been highlighted in Green** throughout the revised manuscript to facilitate verification of the implemented changes.

**Reviewer 3 Comment:** The authors still did not explicitly specify how many samples were collected from a single location, how many extracts were prepared, and how many technical replicates were performed for the biochemical assays.

**Response:** Thank you for this valuable comment. We agree that the description of the sampling and replication strategy was insufficiently detailed in the previous version. Therefore, we have revised the Materials and Methods section to explicitly describe the experimental design. We now state that, for each Crataegus species, fruits and leaves were collected from five healthy and mature individual plants within a single natural population, and these plants were considered biological replicates. Furthermore, three independent methanolic extracts were prepared from each biological replicate, resulting in fifteen extracts per species. All biochemical analyses were subsequently performed in triplicate as technical replicates. In addition, explanatory information regarding the replication structure has been added to the relevant table footnotes. We believe these revisions substantially improve the transparency, reproducibility, and statistical clarity of the study.

**Reviewer 3 Comment:** Generally, the name/model of the HPLC system, detector type, and column manufacturer should be reported. These details are important for researchers who routinely use this technique.

**Response:** Thank you for this valuable suggestion. We agree that detailed instrumental information is important for the reproducibility and transparency of chromatographic analyses. Therefore, we have revised the HPLC methodology section to provide the manufacturer and model of the HPLC system, the detector type and model, and the specifications and manufacturer of the analytical column used in this study. The relevant section now reads:

"Individual phenolic compounds were analyzed using a high-performance liquid chromatography system (Knauer Smartline HPLC, Knauer Wissenschaftliche Geräte GmbH, Berlin, Germany) equipped with a UV–Vis detector (Smartline UV Detector 2500, Knauer Wissenschaftliche Geräte GmbH, Berlin, Germany). Separation was carried out on a Eurospher II 100-5 C18 reverse-phase analytical column (250 × 4.6 mm, 5 μm; Knauer Wissenschaftliche Geräte GmbH, Berlin, Germany) maintained at 25 °C."

These additions improve the methodological clarity and facilitate reproducibility of the analytical procedures.

**Reviewer 3 Comment:** I overlooked when reading the previous version: the molar extinction coefficient (ε, epsilon) has its own unit, most often dm³·mol⁻¹·cm⁻¹. This should be added.

**Response:** Thank you for this valuable comment. We agree that the molar extinction coefficient should be reported together with its appropriate unit. Accordingly, we have revised the Materials and Methods section and added the unit of the molar extinction coefficient used for anthocyanin quantification. The relevant statement now reads:

"ε = 26,900 dm³·mol⁻¹·cm⁻¹ (molar extinction coefficient of cyanidin-3-O-glucoside)."

This revision improves the methodological accuracy and completeness of the anthocyanin determination procedure.

**Regards,**

**Yazgan Tunç, Ph. D.**

**Corresponding author**
