# Supplementary material for: Biochemical characterizations of leaves and fruits in Crataegus monogyna Jacq., C. pontica K.Koch, C. microphylla K.Koch, and C. pentagyna Waldst. & Kit. ex Willd
Source: PLoS One. 2026 Jul 6;21(7):e0352757. doi: 10.1371/journal.pone.0352757 (PMC13336193; doi:10.1371/journal.pone.0352757)
Supplement: Peer review history

# Peer review: Decision Letter 0

**DOI:** 10.1371/journal.pone.0352757.r001

**Authors:** Branislav Šiler

5 May 2026

PONE-D-25-60879

Biochemical characterizations of leaves and fruits in Crataegus monogyna Jacq., C. pontica K.Koch, C. microphylla K.Koch, and C. pentagyna Waldst. & Kit. ex Willd

PLOS One

Dear Dr. Khadivi,

Thank you for submitting your manuscript to PLOS ONE. After careful consideration, we feel that it has merit but does not fully meet PLOS ONE’s publication criteria as it currently stands. Therefore, we invite you to submit a revised version of the manuscript that addresses the points raised during the review process.

==============================

**Two out of three reviewers noted the lack of biological replications during sample collection and metabolite extraction. In such a situation, many results obtained in the study are biased by small sample size and lead to over-explanation by PCA and over-interpretation of correlation and regression analysis. Some methodological errors and inconsistencies were also noted. The text itself is too lengthy in several places, but the discussion section lacks depth and meticulous literature analysis. Finally, the text presentation would benefit from having it proofread by a native English speaker or a professional editing agency. For example, Latin species names are presented under single quotation marks in many places, misleading the reader to present cultivars. Random word capitalization is also noted. L233-235 contain comma to present decimal places. Use 0.989 instead of .989 in Table 5.**

==============================

Please submit your revised manuscript by Jun 19 2026 11:59PM. If you will need more time than this to complete your revisions, please reply to this message or contact the journal office at plosone@plos.org. When you're ready to submit your revision, log on to https://www.editorialmanager.com/pone/ and select the 'Submissions Needing Revision' folder to locate your manuscript file.

Please include the following items when submitting your revised manuscript:

- A letter that responds to each point raised by the academic editor and reviewer(s). You should upload this letter as a separate file labeled 'Response to Reviewers'.
- A marked-up copy of your manuscript that highlights changes made to the original version. You should upload this as a separate file labeled 'Revised Manuscript with Track Changes'.
- An unmarked version of your revised paper without tracked changes. You should upload this as a separate file labeled 'Manuscript'.

If you would like to make changes to your financial disclosure, please include your updated statement in your cover letter. Guidelines for resubmitting your figure files are available below the reviewer comments at the end of this letter.

If applicable, we recommend that you deposit your laboratory protocols in protocols.io to enhance the reproducibility of your results. Protocols.io assigns your protocol its own identifier (DOI) so that it can be cited independently in the future. For instructions see: https://journals.plos.org/plosone/s/submission-guidelines#loc-laboratory-protocols. Additionally, PLOS ONE offers an option for publishing peer-reviewed Lab Protocol articles, which describe protocols hosted on protocols.io. Read more information on sharing protocols at https://plos.org/protocols?utm_medium=editorial-email&utm_source=authorletters&utm_campaign=protocols.

As the corresponding author, your ORCID iD is verified in the submission system and will appear in the published article. PLOS supports the use of ORCID, and we encourage all coauthors to register for an ORCID iD and use it as well. Please encourage your coauthors to verify their ORCID iD within the submission system before final acceptance, as unverified ORCID iDs will not appear in the published article. *Only*  the individual author can complete the verification step; PLOS staff *cannot*  verify ORCID iDs on behalf of authors.

We look forward to receiving your revised manuscript.

Kind regards,

Branislav T. Šiler, Ph.D.

Academic Editor

PLOS One

Journal Requirements:

When submitting your revision, we need you to address these additional requirements.

1. Please ensure that your manuscript meets PLOS ONE's style requirements, including those for file naming. The PLOS ONE style templates can be found at

https://journals.plos.org/plosone/s/file?id=wjVg/PLOSOne_formatting_sample_main_body.pdf and

https://journals.plos.org/plosone/s/file?id=ba62/PLOSOne_formatting_sample_title_authors_affiliations.pdf.

2. We note that your Data Availability Statement is currently as follows: [All relevant data are within the manuscript and its Supporting Information files ]

Please confirm at this time whether or not your submission contains all raw data required to replicate the results of your study. Authors must share the “minimal data set” for their submission. PLOS defines the minimal data set to consist of the data required to replicate all study findings reported in the article, as well as related metadata and methods (https://journals.plos.org/plosone/s/data-availability#loc-minimal-data-set-definition).

For example, authors should submit the following data:

- The values behind the means, standard deviations and other measures reported;

- The values used to build graphs;

- The points extracted from images for analysis.

Authors do not need to submit their entire data set if only a portion of the data was used in the reported study.

If your submission does not contain these data, please either upload them as Supporting Information files or deposit them to a stable, public repository and provide us with the relevant URLs, DOIs, or accession numbers. For a list of recommended repositories, please see https://journals.plos.org/plosone/s/recommended-repositories.

If there are ethical or legal restrictions on sharing a de-identified data set, please explain them in detail (e.g., data contain potentially sensitive information, data are owned by a third-party organization, etc.) and who has imposed them (e.g., an ethics committee). Please also provide contact information for a data access committee, ethics committee, or other institutional body to which data requests may be sent. If data are owned by a third party, please indicate how others may request data access.

3. We note that Figure 1 in your submission contain [map/satellite] images which may be copyrighted. All PLOS content is published under the Creative Commons Attribution License (CC BY 4.0), which means that the manuscript, images, and Supporting Information files will be freely available online, and any third party is permitted to access, download, copy, distribute, and use these materials in any way, even commercially, with proper attribution. For these reasons, we cannot publish previously copyrighted maps or satellite images created using proprietary data, such as Google software (Google Maps, Street View, and Earth). For more information, see our copyright guidelines: http://journals.plos.org/plosone/s/licenses-and-copyright.

We require you to either (1) present written permission from the copyright holder to publish these figures specifically under the CC BY 4.0 license, or (2) remove the figures from your submission:

a. You may seek permission from the original copyright holder of Figure 1 to publish the content specifically under the CC BY 4.0 license.

We recommend that you contact the original copyright holder with the Content Permission Form (http://journals.plos.org/plosone/s/file?id=7c09/content-permission-form.pdf) and the following text:

“I request permission for the open-access journal PLOS ONE to publish XXX under the Creative Commons Attribution License (CCAL) CC BY 4.0 (http://creativecommons.org/licenses/by/4.0/). Please be aware that this license allows unrestricted use and distribution, even commercially, by third parties. Please reply and provide explicit written permission to publish XXX under a CC BY license and complete the attached form.”

Please upload the completed Content Permission Form or other proof of granted permissions as an "Other" file with your submission.

In the figure caption of the copyrighted figure, please include the following text: “Reprinted from [ref] under a CC BY license, with permission from [name of publisher], original copyright [original copyright year].”

b. If you are unable to obtain permission from the original copyright holder to publish these figures under the CC BY 4.0 license or if the copyright holder’s requirements are incompatible with the CC BY 4.0 license, please either i) remove the figure or ii) supply a replacement figure that complies with the CC BY 4.0 license. Please check copyright information on all replacement figures and update the figure caption with source information. If applicable, please specify in the figure caption text when a figure is similar but not identical to the original image and is therefore for illustrative purposes only.

The following resources for replacing copyrighted map figures may be helpful:

USGS National Map Viewer (public domain): http://viewer.nationalmap.gov/viewer/

The Gateway to Astronaut Photography of Earth (public domain): http://eol.jsc.nasa.gov/sseop/clickmap/

Maps at the CIA (public domain): https://www.cia.gov/library/publications/the-world-factbook/index.html and https://www.cia.gov/library/publications/cia-maps-publications/index.html

NASA Earth Observatory (public domain): http://earthobservatory.nasa.gov/

Landsat: http://landsat.visibleearth.nasa.gov/

USGS EROS (Earth Resources Observatory and Science (EROS) Center) (public domain): http://eros.usgs.gov/#

Natural Earth (public domain): http://www.naturalearthdata.com/

4. If the reviewer comments include a recommendation to cite specific previously published works, please review and evaluate these publications to determine whether they are relevant and should be cited. There is no requirement to cite these works unless the editor has indicated otherwise.

[Note: HTML markup is below. Please do not edit.]

Reviewers' comments:

Reviewer's Responses to Questions

**Comments to the Author**

1. Is the manuscript technically sound, and do the data support the conclusions?

The manuscript must describe a technically sound piece of scientific research with data that supports the conclusions. Experiments must have been conducted rigorously, with appropriate controls, replication, and sample sizes. The conclusions must be drawn appropriately based on the data presented.

Reviewer #1: Partly

Reviewer #2: Yes

Reviewer #3: Yes

**********

2. Has the statistical analysis been performed appropriately and rigorously?

Reviewer #1: No

Reviewer #2: Yes

Reviewer #3: I Don't Know

**********

3. Have the authors made all data underlying the findings in their manuscript fully available?

The PLOS Data policy requires authors to make all data underlying the findings described in their manuscript fully available without restriction, with rare exception (please refer to the Data Availability Statement in the manuscript PDF file). The data should be provided as part of the manuscript or its supporting information, or deposited to a public repository. For example, in addition to summary statistics, the data points behind means, medians and variance measures should be available. If there are restrictions on publicly sharing data—e.g. participant privacy or use of data from a third party—those must be specified.

Reviewer #1: No

Reviewer #2: Yes

Reviewer #3: Yes

**********

4. Is the manuscript presented in an intelligible fashion and written in standard English?

PLOS ONE does not copyedit accepted manuscripts, so the language in submitted articles must be clear, correct, and unambiguous. Any typographical or grammatical errors should be corrected at revision, so please note any specific errors here.

Reviewer #1: Yes

Reviewer #2: Yes

Reviewer #3: No

**********

5. Review Comments to the Author

Please use the space provided to explain your answers to the questions above. You may also include additional comments for the author, including concerns about dual publication, research ethics, or publication ethics. (Please upload your review as an attachment if it exceeds 20,000 characters)

Reviewer #1: The manuscript addresses an important and timely topic, namely the comparative biochemical characterization of fruits and leaves of four Crataegus species. The range of analyzed secondary metabolites is broad, and the use of multivariate statistical approaches (PCA, HMA, MRA) represents a clear added value. The study fits well within the scope of PLOS ONE as a descriptive–comparative investigation with potential applied relevance. However, the manuscript suffers from serious methodological, statistical, and interpretative shortcomings, which substantially limit the reliability of several key conclusions in its current form. Major revisions are required before the manuscript can be considered for publication.

1. The writing style is at times overly repetitive and descriptive, particularly in the Results and Discussion section.

Several statements regarding “health benefits” are speculative and go beyond what can be concluded from in vitro antioxidant assays. The separation between results, interpretation, and application-oriented speculation is not always clear.

Some literature citations are discussed superficially, without critical comparison of methodologies.

2. Inconsistencies in units and literature comparisons: The manuscript compares results expressed in:mg/g DW, mg/100 g, mg/kg DW, without consistent conversion. This may mislead readers regarding agreement or disagreement with previous studies.

3. Pseudoreplication and extremely limited biological replication

This is the most serious issue of the study. Each species was collected from only one geographic location. Consequently, species identity is completely confounded with site-specific environmental conditions. As a result: conclusions regarding “species-specific differences” are methodologically unjustified, the application of ANOVA, Pearson correlations, PCA, and MRA with such limited biological replication is statistically problematic. The authors should: clearly state that the study is exploratory or pilot in nature, or restrict their conclusions to “sample-level comparisons” rather than “species-level differentiation”.

4. PCA explaining 100% of variance

A PCA in which three principal components explain exactly 100% of total variance strongly suggests: an extremely small number of observations, strong multicollinearity among variables, potential overparameterization. The manuscript lacks:

explicit information on the number of observations included in PCA,a scree plot, any form of validation of component stability.

In its current form, PCA should be interpreted only as a visualization tool, not as evidence of biologically meaningful differentiation.

5. Overinterpretation of correlations and regression analysis

Correlation coefficients approaching unity (e.g. r = 1.00**) are biologically implausible and most likely reflect: extremely low sample size, definitional or mathematical dependence among variables (e.g. total phenolics vs. phenolic subclasses).

Regarding multiple regression analysis: the ratio of observations to predictors is not reported, no diagnostic tests (normality, multicollinearity, VIF, residual analysis) are provided, the risk of overfitting is very high. Conclusions identifying “key predictors of antioxidant capacity” are therefore not statistically supported.

6. Recommendation: Major revision

The study is based on valuable experimental work and has clear publication potential. However, in its current form it does not meet the statistical and methodological rigor required to support its more ambitious conclusions, particularly those related to species-level differentiation and metabolic regulation. With substantial revisions and a more cautious interpretation of the data, the manuscript could become a solid exploratory contribution to the literature on Crataegus biochemistry.

Reviewer #2: the manuscript is well written and linguistially revised. The refernces are updated. The model is applicable to be taken in consideration and applied in breeding programs thus helping in developing active metabolites for functional food and medicinal applications.

Reviewer #3: This manuscript emphasizes the significance of hawthorn within the Rosaceae family. Despite the widely acknowledged cardioprotective properties of its fruits in numerous countries, the plant itself is frequently underappreciated and treated as a troublesome, thorny weed in temperate forests and scrublands. I consider the subject matter of this study to be valuable and worthy of publication. The manuscript was prepared quite carefully, but it has a few shortcomings. The tables and graphs were prepared with care, and I did not find any serious errors in the analysis of the presented data. Importantly, however, I was unable to find information regarding the number of extracts prepared or the number of replicates for each experiment. Perhaps this information is hidden somewhere, but it should be located in the sections generally designated for this purpose: in the description of the specific methodology, in the section devoted to statistics, or alongside the tables and figures. Without this, it is difficult to consider the presented results and statistical analysis as accurate. I also noticed a few errors and inaccuracies in the methodology. Some parts of the manuscript appear to be written in a rather wordy style and require editorial revisions.

I have compiled my additional comments into a list of points, which I have included below.

1. The authors wrote: ”Chemical properties of four species of Crataegus genus in Iran, including C. monogyna (collected from Dorood area of Lorestan province), C. pontica (collected from Hanivan area of Ilam province), C. microphylla (collected from Gachan area of Ilam province), and C. pentagyna (collected from Babol area of Mazandaran province) were evaluated” and shown the geographic locations on the map. Therefore, the next sentence, i.e. “The plant materials were collected from Lorestan, Ilam, and Mazandaran provinces” seems like an unnecessary repetition. The authors should reorganize Section 2.1.

2. The authors wrote: “After collecting and separating the waste materials, the samples were dried separately at room temperature away from the sunlight in the shade in the airflow and were converted into fine particles with a home grinder and passed through a sieve with 40 mesh.” By crushing the material and sifting it through a sieve, the authors obtain a finely ground material that is not only partially oxidized (which is very difficult to prevent) but also highly susceptible to further oxidation. How did the authors protect it from oxidative degradation? Did they store it in an inert atmosphere at –80 degrees? For how long? It is worth noting that material ground in this way should be used as soon as possible after grinding. The manuscript lacks information on storage.

3. The authors wrote: “All extracts were kept in a refrigerator at 4˚C until analysis.” This is the correct procedure. Although phenolic compound solutions should be stored at a much lower temperature to prevent the degradation of some of the compounds, storing them at 4 degrees Celsius prevents the precipitation of less soluble phenols. The point is that extracts can only be stored this way for a very short time. The authors did not specify exactly how long the extracts were kept at this temperature.

4. In the Folin–Ciocalteu method, the step described as “stopping the reaction after 3 minutes by adding sodium bicarbonate” is of limited significance under the conditions used, since further heating (to 100 degrees Celsius) and prolonged incubation bring the reaction to completion, thereby minimizing the impact of initial time differences.

5. The applied temperature (100°C) may promote partial degradation of thermolabile phenolic compounds, particularly flavonoids, including anthocyanins. The application of a lower temperature (e.g. 40–60°C) may be recommended in future studies to reduce thermal degradation of sensitive phenolics.

6. In the procedure of flavonoid quantification, the authors used ”one µL” of diluted extract.” Isn't there a mistake here? Does the final volume of 2 mL contain 1 μL of diluted sample? In the study cited by the authors (Zhishen et al. 2011), the authors used 500 μL. First of all, that’s not very much; second, mixing reagents in this way (1 + 1999) introduces a scale error.

7. Was the color of the resulting complex actually purple? In the cited study (Zhishen et al. 2011), it was yellow; in others, it was yellow-orange.

8. In the procedure for ortho-diphenol quantification, the authors wrote: “(...) spectrophotometer was zeroed with a blank, and 70% methanol was used as a blank” That is unnecessary repetition. Sentences with a similar structure can also be found in the methods for determining other phenols below.

9. In the determination of hydroxycinnamic acids and flavonols, the primary solvent is 2% hydrochloric acid; therefore, the sample should be zeroed with this solution, not with 70% methanol.

10. The sentence in line 183, i.e. “Put the red number in equation 1 and get the absorption rate of the diluted sample” should be removed.

11. The authors wrote:” The ability to remove DPPH of the samples was evaluated by the method proposed by Obied et al. [19] with minor changes”. This is a rather unfortunate (and incorrect) interpretation of the principle behind this measurement. DPPH radical is not removed but scavenged or simpler: reduced. I would also avoid using the incorrect term “DPPH removal.” (line 198).

12. A DPPH solution in methanol is purple, not blue (line 193). The color changes from purple through reddish to pale yellow. Therefore, the notation “purple → yellow to red” incorrectly indicates the direction of the change.

13. The HPLC methodology needs to be rewritten from scratch. I do not understand what is meant by ‘the calibration curve (...) was drawn at a wavelength of 220 to 400 nm’. This is an oversimplification. The description does not provide a detailed stepwise gradient profile, the total separation time, and the wavelength used for separating the test samples. There are no specifications for the column, chromatograph or detector. The authors have not stated where they obtained the standards. Analysis of the text does not prove that these were high-purity compounds.

14. There is an error in the description of the filter size, i.e. ‘0.2 mm syringe filter’. I believe it should have been written as ‘0.20 micrometers’.

15. In the description of the statistical analysis, specifically Pearson’s correlation – the significance level (I assume this is also p = 0.005) should also be stated.

16. In the description of the statistical analysis, the type of test is missing from the description of the one-way ANOVA, and in the description of Pearson’s correlation, the significance level (I believe also p = 0.05) should also be supplemented.

17. In line 236, the authors uses two the same phrases: “antioxidant capacity (5,80), and antioxidant capacity (1,26)”. A word (fruit/leaf) or symbol should appear here to distinguish between these two items.

18. In the text between lines 232 and 237, the numerical values in parentheses should be followed by a percentage symbol. Generally, when presenting values one after another, at least the first set of parentheses should include the percent sign. Parentheses containing numbers without percentages appear later in the Results section.

19. The authors wrote: “The biochemical contents of some Crataegus genus fruits are presented in Table 2.” This sentence is misleading because a plant’s “biochemical content” primarily refers to its essential components, and none are mentioned here. The next sentence clarifies the meaning and, in my opinion, is sufficient. I would omit the quoted sentence. Moreover, I would modify the title of Table 2 from “Biochemical contents of some Crataegus genus fruits” to “Phytochemical contents of some Crataegus genus fruits”.

20. The text between lines 254 and 278 essentially describes what we can see directly in Table 2. In my opinion, such a detailed description is unnecessary; the text here should be more concise.

21. The authors wrote: “In PCA, the original variables are transformed into a smaller set of uncorrelated components, known as principal components, that explain the maximum variance in the data. This method is widely employed for data reduction, pattern recognition, and identifying underlying structures in complex datasets [53]”. In my opinion there is no need to describe what PCA is as PCA’s are widely uses in recent scientific literature. This text should be condensed.

22. The following paragraphs on standardization and the details of PCA are helpful, as this information is not widely known. I would just suggest shortening them slightly.

23. I would advise the authors to describe other statistical tools (e.g., correlation matrix analysis) more concisely.

24. In Figures 2 and 3, the values shown in pale pink are almost invisible.

25. The p-values listed below Figures 2 and 3 should be written in italics.

26. The conclusion section should be no longer than 5–6 sentences, excluding the “introduction”. This one has 11 sentences and is too descriptive in the first part; it resembles a standard abstract too closely.

27. The authors may consider replacing hyphens (-) with en dashes (–) or the proper minus sign (−) in the tables and the regular text, where negative values are indicated, to maintain typographical consistency.

28. There should be a space before and after the equal sign (including when authors cite values in the text).

May 1, 2026

**********

6. PLOS authors have the option to publish the peer review history of their article (what does this mean?). If published, this will include your full peer review and any attached files.

If you choose “no”, your identity will remain anonymous but your review may still be made public.

**Do you want your identity to be public for this peer review?**  For information about this choice, including consent withdrawal, please see our Privacy Policy.

Reviewer #1: No

Reviewer #2: No

Reviewer #3: No

**********

[NOTE: If reviewer comments were submitted as an attachment file, they will be attached to this email and accessible via the submission site. Please log into your account, locate the manuscript record, and check for the action link "View Attachments". If this link does not appear, there are no attachment files.]

To ensure your figures meet our technical requirements, please review our figure guidelines: https://journals.plos.org/plosone/s/figures

You may also use PLOS’s free figure tool, NAAS, to help you prepare publication quality figures: https://journals.plos.org/plosone/s/figures#loc-tools-for-figure-preparation.

NAAS will assess whether your figures meet our technical requirements by comparing each figure against our figure specifications.

# Author comment: Author response to Decision Letter 1

**DOI:** 10.1371/journal.pone.0352757.r002

17 May 2026

Dear Editor,

Thank you very much for your valuable comments, criticisms, and suggestions regarding our manuscript. In accordance with your recommendations, the manuscript has been carefully revised throughout.

The concerns related to limited biological replication, PCA interpretation, correlation and regression analyses, methodological inconsistencies, and statistical overinterpretation were carefully reconsidered, and the relevant sections were revised with a more cautious and scientifically balanced interpretation. In addition, the Discussion section was substantially improved with a deeper and more meticulous literature evaluation.

The manuscript text was also revised to reduce repetitive expressions and improve overall clarity and readability. Problems related to species nomenclature, random capitalization, decimal notation, and numerical formatting were corrected throughout the manuscript, including the correction of “.989” to “0.989” in Table 5.

Furthermore, the entire manuscript underwent professional English language editing using Grammarly (https://www.grammarly.com) to improve grammar, readability, sentence structure, and overall linguistic quality.

For easier tracking of the revisions within the revised manuscript, modifications made in response to the Editor’s comments were highlighted in yellow, revisions corresponding to Reviewer #1 comments were highlighted in green, and revisions related to Reviewer #3 comments were highlighted in turquoise directly in the manuscript text.

We sincerely appreciate the Editor’s careful evaluation and constructive guidance, which significantly improved the scientific quality and presentation of our manuscript.

Kind regards,

Dear Reviewer #1:

We sincerely thank Reviewer #1 for the valuable time devoted to evaluating our manuscript and for the constructive comments and suggestions, which have significantly contributed to improving the scientific quality and clarity of the study. All comments and recommendations were carefully considered and addressed in the revised manuscript. To facilitate the review process and allow the changes to be followed more easily, all revisions made in response to Reviewer #1’s comments have been highlighted in green in the manuscript.

Kind regards,

Reviewer #1, comment 1: The writing style is at times overly repetitive and descriptive, particularly in the Results and Discussion section. Several statements regarding “health benefits” are speculative and go beyond what can be concluded from in vitro antioxidant assays. The separation between results, interpretation, and application-oriented speculation is not always clear. Some literature citations are discussed superficially, without critical comparison of methodologies.

Response to Reviewer #1, comment 1: We sincerely thank the reviewer for this valuable comment. In accordance with the criticism and suggestions provided, the Results and Discussion section was carefully revised to reduce repetitive and overly descriptive expressions. Statements related to potential health benefits were moderated and restricted to conclusions directly supported by the in vitro antioxidant assays. In addition, the distinction between experimental findings, interpretations, and application-oriented implications was clarified throughout the manuscript. Relevant literature comparisons were also strengthened by including a more critical evaluation of methodological differences among previous studies.

Reviewer #1, comment 2: Inconsistencies in units and literature comparisons: The manuscript compares results expressed in:mg/g DW, mg/100 g, mg/kg DW, without consistent conversion. This may mislead readers regarding agreement or disagreement with previous studies.

Response to Reviewer #1, comment 2: We thank the reviewer for this important comment. The units used for the biochemical data in the present study were checked carefully and were confirmed to be correct. However, in accordance with the reviewer’s concern, the literature comparison sections were revised to avoid any misleading direct comparison among studies using different reporting units, such as mg/g DW, mg/100 g, mg/kg DW, FW-based expressions, or different antioxidant assay units. We clarified that comparisons with previous studies should be interpreted cautiously unless the same dry-weight basis, extraction procedure, analytical method, and reporting unit were used. In addition, the wording of the discussion was revised by replacing direct “higher/lower” or “comparable” statements with more cautious expressions emphasizing methodological and unit-based differences among studies.

Reviewer #1, comment 3: Pseudoreplication and extremely limited biological replication

This is the most serious issue of the study. Each species was collected from only one geographic location. Consequently, species identity is completely confounded with site-specific environmental conditions. As a result: conclusions regarding “species-specific differences” are methodologically unjustified, the application of ANOVA, Pearson correlations, PCA, and MRA with such limited biological replication is statistically problematic. The authors should: clearly state that the study is exploratory or pilot in nature, or restrict their conclusions to “sample-level comparisons” rather than “species-level differentiation”.

Response to Reviewer #1, comment 3: We thank the reviewer for this important methodological observation. We acknowledge that each Crataegus species in the present study was represented by samples collected from a single geographic location, which may confound species-related and environmental effects. In accordance with the reviewer’s suggestion, the manuscript was revised to clarify the exploratory nature of the study. Statements implying definitive species-level differentiation were moderated throughout the manuscript and replaced with more cautious expressions referring to sample-level biochemical variation among the analyzed materials. In addition, limitations related to biological replication and environmental confounding were explicitly acknowledged in the Materials and Methods, Results and Discussion, and Conclusion sections. The interpretations of ANOVA, PCA, correlation, and regression analyses were also revised to emphasize their descriptive and exploratory purpose within the present dataset rather than definitive species-wide generalizations.

Reviewer #1, comment 4: PCA explaining 100% of variance A PCA in which three principal components explain exactly 100% of total variance strongly suggests: an extremely small number of observations, strong multicollinearity among variables, potential overparameterization. The manuscript lacks: explicit information on the number of observations included in PCA,a scree plot, any form of validation of component stability. In its current form, PCA should be interpreted only as a visualization tool, not as evidence of biologically meaningful differentiation.

Response to Reviewer #1, comment 4: We thank the reviewer for this valuable and technically important comment. We acknowledge that the relatively limited number of analyzed accessions may influence the variance structure observed in the PCA. In the present dataset, four sampled Crataegus materials were evaluated; therefore, the extraction of three principal components collectively explaining 100% of the variance is mathematically expected in PCA applications involving a small number of observations.

In accordance with the reviewer’s suggestion, the interpretation of the PCA was revised throughout the manuscript to emphasize its exploratory and descriptive nature rather than presenting it as definitive evidence of biologically meaningful species-level differentiation. Additional clarification regarding the number of observations included in the PCA was added to the Materials and Methods section, and the PCA discussion was carefully moderated using more cautious terminology.

Furthermore, the PCA was primarily employed as a multivariate visualization approach to explore relationships among biochemical variables and sampled materials within the present dataset. We agree that the observed clustering patterns should be interpreted cautiously due to the limited biological replication and the potential influence of multicollinearity among variables. Accordingly, statements implying definitive biological separation or metabolic regulation were revised throughout the manuscript.

In addition, an exploratory PCA score plot was added as Supplementary Figure S1 to improve the visualization and transparency of the multivariate analysis.

Reviewer #1, comment 5: Overinterpretation of correlations and regression analysis Correlation coefficients approaching unity (e.g. r = 1.00**) are biologically implausible and most likely reflect: extremely low sample size, definitional or mathematical dependence among variables (e.g. total phenolics vs. phenolic subclasses). Regarding multiple regression analysis: the ratio of observations to predictors is not reported, no diagnostic tests (normality, multicollinearity, VIF, residual analysis) are provided, the risk of overfitting is very high. Conclusions identifying “key predictors of antioxidant capacity” are therefore not statistically supported.

Response to Reviewer #1, comment 5: We thank the reviewer for this valuable statistical comment. We acknowledge that the limited number of analyzed samples may contribute to inflated correlation coefficients and increase the risk of overfitting in multivariate statistical analyses. In accordance with the reviewer’s suggestions, the interpretations of the correlation matrix analysis and multiple regression analysis were carefully revised throughout the manuscript to avoid overly definitive biological conclusions.

Reviewer #2:

The manuscript is well written and linguistially revised. The refernces are updated. The model is applicable to be taken in consideration and applied in breeding programs thus helping in developing active metabolites for functional food and medicinal applications.

Dear Reviewer #2:

We sincerely thank Reviewer #2 for the valuable time devoted to evaluating our manuscript and for the positive and constructive comments. We are pleased that the linguistic quality of the manuscript, the updated references, and the potential applicability of the study in breeding programs as well as functional food and medicinal applications were appreciated. The reviewer’s encouraging evaluation and valuable remarks have significantly contributed to strengthening the scientific quality of our study.

Kind regards,

Dear Reviewer #3:

We sincerely thank Reviewer #3 for the valuable time and effort devoted to the careful evaluation of our manuscript. The insightful comments and constructive suggestions have significantly contributed to improving the scientific quality and clarity of the study. In accordance with the reviewer’s recommendations, all necessary revisions were carefully addressed and incorporated into the revised manuscript. For the convenience of the reviewer and to facilitate easier tracking of the modifications, all changes made in response to Reviewer #3’s comments have been highlighted in turquoise color throughout the manuscript.

Kind regards,

Reviewer #3, comment 1: The authors wrote: ”Chemical properties of four species of Crataegus genus in Iran, including C. monogyna (collected from Dorood area of Lorestan province), C. pontica (collected from Hanivan area of Ilam province), C. microphylla (collected from Gachan area of Ilam province), and C. pentagyna (collected from Babol area of Mazandaran province) were evaluated” and shown the geographic locations on the map. Therefore, the next sentence, i.e. “The plant materials were collected from Lorestan, Ilam, and Mazandaran provinces” seems like an unnecessary repetition. The authors should reorganize Section 2.1.

Response to Reviewer #3, comment 1: Thank you for your criticism and suggestions. The relevant text has been corrected.

Reviewer #3, comment 2: The authors wrote: “After collecting and separating the waste materials, the samples were dried separately at room temperature away from the sunlight in the shade in the airflow and were converted into fine particles with a home grinder and passed through a sieve with 40 mesh.” By crushing the material and sifting it through a sieve, the authors obtain a finely ground material that is not only partially oxidized (which is very difficult to prevent) but also highly susceptible to further oxidation. How did the authors protect it from oxidative degradation? Did they store it in an inert atmosphere at –80 degrees? For how long? It is worth noting that material ground in this way should be used as soon as possible after grinding. The manuscript lacks information on storage.

Response to Reviewer #3, comment 2: We sincerely thank the reviewer for this valuable and scientifically important comment regarding the potential oxidative degradation of finely ground plant materials. We fully agree that powdered plant samples are highly susceptible to oxidation after grinding and sieving procedures, particularly in studies focusing on phenolic compounds and antioxidant-related traits.

To address this concern, additional methodological details have now been incorporated into the revised manuscript. Specifically, after grinding and sieving, the powdered samples were immediately transferred into airtight amber glass containers protected from light and air exposure. The samples were subsequently stored at −20 °C for no longer than 72 h before extraction and were subjected to methanolic extraction as soon as possible after grinding to minimize oxidative degradation of phenolic compounds.

These clarifications have been added to the Materials and Methods section of the revised manuscript.

Reviewer #3, comment 3: The authors wrote: “All extracts were kept in a refrigerator at 4˚C until analysis.” This is the correct procedure. Although phenolic compound solutions should be stored at a much lower temperature to prevent the degradation of some of the compounds, storing them at 4 degrees Celsius prevents the precipitation of less soluble phenols. The point is that extracts can only be stored this way for a very short time. The authors did not specify exactly how long the extracts were kept at this temperature.

Response to Reviewer #3, comment 3: We sincerely thank the Reviewer for this valuable and scientifically important comment. In accordance with the suggestion, we clarified the storage duration of the extracts in the revised manuscript. The text was revised to indicate that all extracts were stored in amber glass containers at 4 ˚C and analyzed within 24 h after extraction in order to minimize oxidative and biochemical degradation of phenolic compounds while preventing precipitation of less soluble phenolics. This clarification has been incorporated into Section 2.2 (“Chemical evaluations”) of the revised manuscript.

Reviewer #3, comment 4: “In the Folin–Ciocalteu method, the step described as ‘stopping the reaction after 3 minutes by adding sodium bicarbonate’ is of limited significance under the conditions used, since further heating (to 100°C) and prolonged incubation bring the reaction to completion, thereby minimizing the impact of initial time differences.

Reviewer #3, comment 5: The applied temperature (100°C) may promote partial degradation of thermolabile phenolic compounds, particularly flavonoids, including anthocyanins. The application of a lower temperature (e.g. 40–60°C) may be recommended in future studies to reduce thermal degradation of sensitive phenolics.”

Response to Reviewer #3, comment 4 and 5: We sincerely thank the Reviewer for this valuable and scientifically important comment. In We sincerely thank the Reviewer for this valuable methodological comment. In accordance with the suggestion, the phrase “stopping the reaction” was revised to more accurately reflect the role of sodium bicarbonate in providing alkaline conditions necessary for color development in the Folin–Ciocalteu assay. In addition, we clarified that the subsequent heating and incubation steps were intended to allow completion of the reaction.

We also acknowledge the Reviewer’s important observation regarding the pos

Attachment Submitted filename: Response to Reviewers.docx

# Peer review: Decision Letter 1

**DOI:** 10.1371/journal.pone.0352757.r003

**Authors:** Branislav Šiler, Branislav Šiler

8 Jun 2026

PONE-D-25-60879R1

Biochemical characterizations of leaves and fruits in Crataegus monogyna Jacq., C. pontica K.Koch, C. microphylla K.Koch, and C. pentagyna Waldst. & Kit. ex Willd

PLOS One

Dear Dr. Khadivi,

Thank you for submitting your manuscript to PLOS ONE. After careful consideration, we feel that it has merit but does not fully meet PLOS ONE’s publication criteria as it currently stands. Therefore, we invite you to submit a revised version of the manuscript that addresses the points raised during the review process.

Please submit your revised manuscript by Jul 23 2026 11:59PM. If you will need more time than this to complete your revisions, please reply to this message or contact the journal office at plosone@plos.org. When you're ready to submit your revision, log on to https://www.editorialmanager.com/pone/ and select the 'Submissions Needing Revision' folder to locate your manuscript file.

Please include the following items when submitting your revised manuscript:

- A letter that responds to each point raised by the academic editor and reviewer(s). You should upload this letter as a separate file labeled 'Response to Reviewers'.
- A marked-up copy of your manuscript that highlights changes made to the original version. You should upload this as a separate file labeled 'Revised Manuscript with Track Changes'.
- An unmarked version of your revised paper without tracked changes. You should upload this as a separate file labeled 'Manuscript'.

If you would like to make changes to your financial disclosure, please include your updated statement in your cover letter. Guidelines for resubmitting your figure files are available below the reviewer comments at the end of this letter.

If applicable, we recommend that you deposit your laboratory protocols in protocols.io to enhance the reproducibility of your results. Protocols.io assigns your protocol its own identifier (DOI) so that it can be cited independently in the future. For instructions see: https://journals.plos.org/plosone/s/submission-guidelines#loc-laboratory-protocols. Additionally, PLOS ONE offers an option for publishing peer-reviewed Lab Protocol articles, which describe protocols hosted on protocols.io. Read more information on sharing protocols at https://plos.org/protocols?utm_medium=editorial-email&utm_source=authorletters&utm_campaign=protocols.

As the corresponding author, your ORCID iD is verified in the submission system and will appear in the published article. PLOS supports the use of ORCID, and we encourage all coauthors to register for an ORCID iD and use it as well. Please encourage your coauthors to verify their ORCID iD within the submission system before final acceptance, as unverified ORCID iDs will not appear in the published article. *Only*  the individual author can complete the verification step; PLOS staff *cannot*  verify ORCID iDs on behalf of authors.

We look forward to receiving your revised manuscript.

Kind regards,

Branislav T. Šiler, Ph.D.

Academic Editor

PLOS One

Journal Requirements:

1. If the reviewer comments include a recommendation to cite specific previously published works, please review and evaluate these publications to determine whether they are relevant and should be cited. There is no requirement to cite these works unless the editor has indicated otherwise.

Additional Editor Comments:

**The sampling procedure has remained insubstantially described (please see the Reviewer's comments below). A detailed sampling protocol should be submitted. Fig 2 and Fig 3 still contain comma instead full stop to define decimal places. Please correct.**

[Note: HTML markup is below. Please do not edit.]

Reviewer's Responses to Questions

**Comments to the Author**

1. If the authors have adequately addressed your comments raised in a previous round of review and you feel that this manuscript is now acceptable for publication, you may indicate that here to bypass the “Comments to the Author” section, enter your conflict of interest statement in the “Confidential to Editor” section, and submit your "Accept" recommendation.

Reviewer #3: (No Response)

**********

2. Is the manuscript technically sound, and do the data support the conclusions?

The manuscript must describe a technically sound piece of scientific research with data that supports the conclusions. Experiments must have been conducted rigorously, with appropriate controls, replication, and sample sizes. The conclusions must be drawn appropriately based on the data presented.

Reviewer #3: Partly

**********

3. Has the statistical analysis been performed appropriately and rigorously?

Reviewer #3: I Don't Know

**********

4. Have the authors made all data underlying the findings in their manuscript fully available?

The PLOS Data policy requires authors to make all data underlying the findings described in their manuscript fully available without restriction, with rare exception (please refer to the Data Availability Statement in the manuscript PDF file). The data should be provided as part of the manuscript or its supporting information, or deposited to a public repository. For example, in addition to summary statistics, the data points behind means, medians and variance measures should be available. If there are restrictions on publicly sharing data—e.g. participant privacy or use of data from a third party—those must be specified.

Reviewer #3: Yes

**********

5. Is the manuscript presented in an intelligible fashion and written in standard English?

PLOS ONE does not copyedit accepted manuscripts, so the language in submitted articles must be clear, correct, and unambiguous. Any typographical or grammatical errors should be corrected at revision, so please note any specific errors here.

Reviewer #3: Yes

**********

6. Review Comments to the Author

Please use the space provided to explain your answers to the questions above. You may also include additional comments for the author, including concerns about dual publication, research ethics, or publication ethics. (Please upload your review as an attachment if it exceeds 20,000 characters)

Reviewer #3: In my opinion, a very important point raised in the opening sentences of my review was not fully addressed. Like Reviewer 1, I pointed out deficiencies in the statistics: “Importantly, however, I was unable to find information regarding the number of extracts prepared or the number of replicates for each experiment. Perhaps this information is hidden somewhere, but it should be located in the sections generally designated for this purpose: in the description of the specific methodology, in the section devoted to statistics, or alongside the tables and figures. Without this, it is difficult to consider the presented results and statistical analysis as accurate”.

The authors included a sentence in the revised version: “Because each species was sampled from a single geographic location, species identity and environmental conditions may not be completely independent. Therefore, the present dataset primarily reflects sample-level biochemical variation under the specific ecological conditions of the collection sites”. I understand that there is only one sampling site. However, the authors still did not explicitly specify how many samples they collected from a single location, how many extracts they prepared, and in how many technical replicates they performed the individual biochemical assays. In Table 1, they provided standard deviations for all results, so the number of replicates, n, should be given below the table. This is a serious omission.

Most of my other comments have been incorporated, and the text has been modified and expanded. In my opinion, the revised manuscript is more substantive and more carefully presented. I have only two minor comments.

1. Typically, the name of the high-performance liquid chromatograph, the detector type, and the column manufacturer are provided. For those who use this technique on a daily basis, this information is essential.

2. I overlooked when reading the previous version: the molar extinction coefficient, epsilon, has its own unit, most often: dm³·mol⁻¹·cm⁻¹ (line 242). This should be added.

May 20, 2026

**********

7. PLOS authors have the option to publish the peer review history of their article (what does this mean?). If published, this will include your full peer review and any attached files.

If you choose “no”, your identity will remain anonymous but your review may still be made public.

**Do you want your identity to be public for this peer review?**  For information about this choice, including consent withdrawal, please see our Privacy Policy.

Reviewer #3: No

**********

[NOTE: If reviewer comments were submitted as an attachment file, they will be attached to this email and accessible via the submission site. Please log into your account, locate the manuscript record, and check for the action link "View Attachments". If this link does not appear, there are no attachment files.]

To ensure your figures meet our technical requirements, please review our figure guidelines: https://journals.plos.org/plosone/s/figures

You may also use PLOS’s free figure tool, NAAS, to help you prepare publication quality figures: https://journals.plos.org/plosone/s/figures#loc-tools-for-figure-preparation.

NAAS will assess whether your figures meet our technical requirements by comparing each figure against our figure specifications.

# Author comment: Author response to Decision Letter 2

**DOI:** 10.1371/journal.pone.0352757.r004

8 Jun 2026

Dear Editor,

We sincerely thank the Editor for the time, effort, and valuable comments devoted to the evaluation of our manuscript. The constructive suggestions and recommendations have significantly improved the scientific quality, clarity, and overall presentation of the manuscript. We have carefully addressed all comments and implemented the requested revisions throughout the manuscript. To facilitate the review process and enable easy identification of the modifications, all changes made in response to the Editor’s comments have been highlighted in Yellow in the revised manuscript.

Editor Comment: Fig. 2 and Fig. 3 still contain comma instead full stop to define decimal places. Please correct.

Response: Thank you for your careful observation. We have thoroughly revised the manuscript and corrected all decimal separators throughout the document. In addition, the former Figure 2 and Figure 3 have been reorganized and are now presented as Table 5 and Table 6, respectively. All decimal values have been standardized using full stops (.) instead of commas (,), in accordance with the journal's formatting requirements. The revised tables have been carefully checked to ensure consistency and accuracy throughout the manuscript.

Regards,

Dear Reviewer 3,

We would like to express our sincere gratitude to Reviewer 3 for the careful evaluation of our manuscript and for the insightful and constructive comments provided. The reviewer’s suggestions have been extremely valuable in improving the methodological clarity, transparency, and scientific rigor of the study. We have carefully considered all comments and revised the manuscript accordingly. For the convenience of the reviewer, all modifications made in response to Reviewer 3’s comments have been highlighted in Green throughout the revised manuscript to facilitate verification of the implemented changes.

Reviewer 3 Comment: The authors still did not explicitly specify how many samples were collected from a single location, how many extracts were prepared, and how many technical replicates were performed for the biochemical assays.

Response: Thank you for this valuable comment. We agree that the description of the sampling and replication strategy was insufficiently detailed in the previous version. Therefore, we have revised the Materials and Methods section to explicitly describe the experimental design. We now state that, for each Crataegus species, fruits and leaves were collected from five healthy and mature individual plants within a single natural population, and these plants were considered biological replicates. Furthermore, three independent methanolic extracts were prepared from each biological replicate, resulting in fifteen extracts per species. All biochemical analyses were subsequently performed in triplicate as technical replicates. In addition, explanatory information regarding the replication structure has been added to the relevant table footnotes. We believe these revisions substantially improve the transparency, reproducibility, and statistical clarity of the study.

Reviewer 3 Comment: Generally, the name/model of the HPLC system, detector type, and column manufacturer should be reported. These details are important for researchers who routinely use this technique.

Response: Thank you for this valuable suggestion. We agree that detailed instrumental information is important for the reproducibility and transparency of chromatographic analyses. Therefore, we have revised the HPLC methodology section to provide the manufacturer and model of the HPLC system, the detector type and model, and the specifications and manufacturer of the analytical column used in this study. The relevant section now reads:

"Individual phenolic compounds were analyzed using a high-performance liquid chromatography system (Knauer Smartline HPLC, Knauer Wissenschaftliche Geräte GmbH, Berlin, Germany) equipped with a UV–Vis detector (Smartline UV Detector 2500, Knauer Wissenschaftliche Geräte GmbH, Berlin, Germany). Separation was carried out on a Eurospher II 100-5 C18 reverse-phase analytical column (250 × 4.6 mm, 5 μm; Knauer Wissenschaftliche Geräte GmbH, Berlin, Germany) maintained at 25 °C."

These additions improve the methodological clarity and facilitate reproducibility of the analytical procedures.

Reviewer 3 Comment: I overlooked when reading the previous version: the molar extinction coefficient (ε, epsilon) has its own unit, most often dm³·mol⁻¹·cm⁻¹. This should be added.

Response: Thank you for this valuable comment. We agree that the molar extinction coefficient should be reported together with its appropriate unit. Accordingly, we have revised the Materials and Methods section and added the unit of the molar extinction coefficient used for anthocyanin quantification. The relevant statement now reads:

"ε = 26,900 dm³·mol⁻¹·cm⁻¹ (molar extinction coefficient of cyanidin-3-O-glucoside)."

This revision improves the methodological accuracy and completeness of the anthocyanin determination procedure.

Regards,

Yazgan Tunç, Ph. D.

Corresponding author

Attachment Submitted filename: Responses to the Editor and Reviewer 3.docx

# Peer review: Decision Letter 2

**DOI:** 10.1371/journal.pone.0352757.r005

**Authors:** Branislav Šiler, Branislav Šiler, Branislav Šiler

16 Jun 2026

Biochemical characterizations of leaves and fruits in Crataegus monogyna Jacq., C. pontica K.Koch, C. microphylla K.Koch, and C. pentagyna Waldst. & Kit. ex Willd

PONE-D-25-60879R2

Dear Dr. Khadivi,

We’re pleased to inform you that your manuscript has been judged scientifically suitable for publication and will be formally accepted for publication once it meets all outstanding technical requirements.

Within one week, you’ll receive an e-mail detailing the required amendments. When these have been addressed, you’ll receive a formal acceptance letter and your manuscript will be scheduled for publication.

An invoice will be generated when your article is formally accepted. Please note, if your institution has a publishing partnership with PLOS and your article meets the relevant criteria, all or part of your publication costs will be covered. Please make sure your user information is up-to-date by logging into Editorial Manager at Editorial Manager® and clicking the ‘Update My Information' link at the top of the page. For questions related to billing, please contact billing support.

If your institution or institutions have a press office, please notify them about your upcoming paper to help maximize its impact. If they’ll be preparing press materials, please inform our press team as soon as possible -- no later than 48 hours after receiving the formal acceptance. Your manuscript will remain under strict press embargo until 2 pm Eastern Time on the date of publication. For more information, please contact onepress@plos.org.

Kind regards,

Branislav T. Šiler, Ph.D.

Academic Editor

PLOS One

Additional Editor Comments (optional):

Reviewers' comments:

Reviewer's Responses to Questions

**Comments to the Author**

1. If the authors have adequately addressed your comments raised in a previous round of review and you feel that this manuscript is now acceptable for publication, you may indicate that here to bypass the “Comments to the Author” section, enter your conflict of interest statement in the “Confidential to Editor” section, and submit your "Accept" recommendation.

Reviewer #3: All comments have been addressed

**********

2. Is the manuscript technically sound, and do the data support the conclusions?

The manuscript must describe a technically sound piece of scientific research with data that supports the conclusions. Experiments must have been conducted rigorously, with appropriate controls, replication, and sample sizes. The conclusions must be drawn appropriately based on the data presented.

Reviewer #3: Yes

**********

3. Has the statistical analysis been performed appropriately and rigorously?

Reviewer #3: Yes

**********

4. Have the authors made all data underlying the findings in their manuscript fully available?

The PLOS Data policy requires authors to make all data underlying the findings described in their manuscript fully available without restriction, with rare exception (please refer to the Data Availability Statement in the manuscript PDF file). The data should be provided as part of the manuscript or its supporting information, or deposited to a public repository. For example, in addition to summary statistics, the data points behind means, medians and variance measures should be available. If there are restrictions on publicly sharing data—e.g. participant privacy or use of data from a third party—those must be specified.

Reviewer #3: Yes

**********

5. Is the manuscript presented in an intelligible fashion and written in standard English?

PLOS ONE does not copyedit accepted manuscripts, so the language in submitted articles must be clear, correct, and unambiguous. Any typographical or grammatical errors should be corrected at revision, so please note any specific errors here.

Reviewer #3: Yes

**********

6. Review Comments to the Author

Please use the space provided to explain your answers to the questions above. You may also include additional comments for the author, including concerns about dual publication, research ethics, or publication ethics. (Please upload your review as an attachment if it exceeds 20,000 characters)

Reviewer #3: The authors have addressed all of my comments. The number of replicates is now specified not only in the “Materials and Methods” section but also listed below the tables, which enhances the clarity of the results. As I suggested, the authors have provided a comprehensive description of the chromatograph used and its accessories. I have no further substantive comments regarding the manuscript. In my opinion, it can proceed to publication.

**********

7. PLOS authors have the option to publish the peer review history of their article (what does this mean?). If published, this will include your full peer review and any attached files.

If you choose “no”, your identity will remain anonymous but your review may still be made public.

**Do you want your identity to be public for this peer review?**  For information about this choice, including consent withdrawal, please see our Privacy Policy.

Reviewer #3: No

**********

# Editor report: Acceptance letter

**DOI:** 10.1371/journal.pone.0352757.r006

**Authors:** Branislav Šiler, Branislav Šiler, Branislav Šiler

PONE-D-25-60879R2

PLOS One

Dear Dr. Khadivi,

I'm pleased to inform you that your manuscript has been deemed suitable for publication in PLOS One. Congratulations! Your manuscript is now being handed over to our production team.

At this stage, our production department will prepare your paper for publication. This includes ensuring the following:

* All references, tables, and figures are properly cited

* All relevant supporting information is included in the manuscript submission,

* There are no issues that prevent the paper from being properly typeset

You will receive further instructions from the production team, including instructions on how to review your proof when it is ready. Please keep in mind that we are working through a large volume of accepted articles, so please give us a few days to review your paper and let you know the next and final steps.

Lastly, if your institution or institutions have a press office, please let them know about your upcoming paper now to help maximize its impact. If they'll be preparing press materials, please inform our press team within the next 48 hours. Your manuscript will remain under strict press embargo until 2 pm Eastern Time on the date of publication. For more information, please contact onepress@plos.org.

You will receive an invoice from PLOS for your publication fee after your manuscript has reached the completed accept phase. If you receive an email requesting payment before acceptance or for any other service, this may be a phishing scheme. Learn how to identify phishing emails and protect your accounts at https://explore.plos.org/phishing.

If we can help with anything else, please email us at customercare@plos.org.

Thank you for submitting your work to PLOS ONE and supporting open access.

Kind regards,

PLOS ONE Editorial Office Staff

on behalf of

Dr. Branislav T. Šiler

Academic Editor

PLOS One
